# Supplementary material for: Cross-species dissection of saline-related genes by genetically deciphering a euryhaline microalga Chlorella sp
Source: Nat Commun. 2026 Jan 13;17:1577. doi: 10.1038/s41467-026-68287-6 (PMC12902096; doi:10.1038/s41467-026-68287-6)
Supplement: Supplementary file 1 — Supplementary Information [file 41467_2026_68287_MOESM1_ESM.pdf]

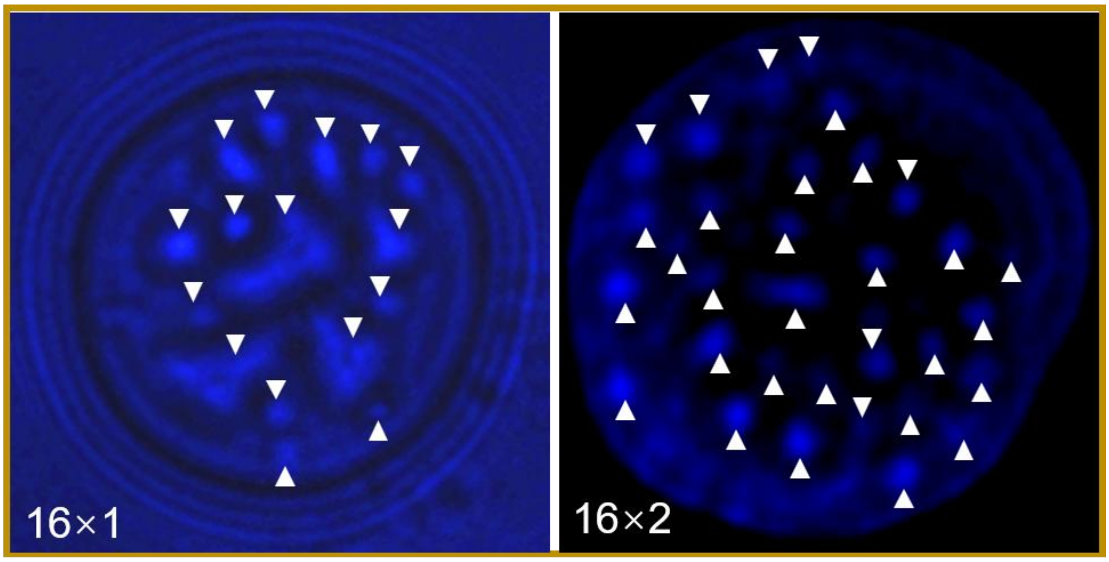

Fig. S1. Enumeration of chromosomes revealed by 4’,6-diamidino-2-phenylindole (DAPI) staining. The white triangles mark the chromosomes. All source data are provided as a Source Data file.

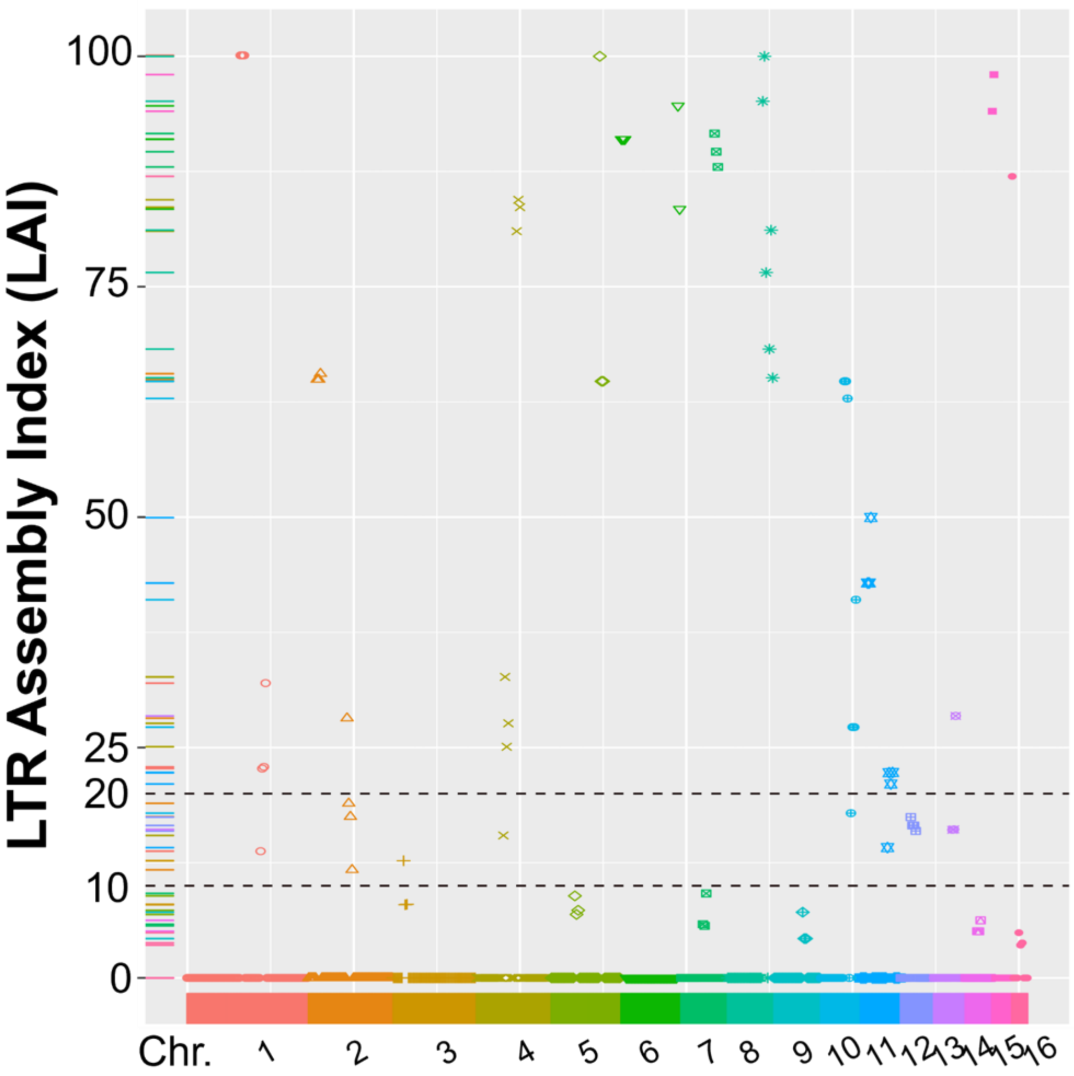

Fig. S2. Long Terminal Repeat Assembly Index (LAI) of *Chlorella* sp. MEM25 genome. The X-axis indicates sequence region of the 16 chromosomes in different colors, with a resolution of 100 kb. The Y-axis denotes the LAI value (LAI = 100 × Intact Long Terminal Repeat retrotransposon length / Total Long Terminal Repeat sequence length). All source data are provided as a Source Data file.

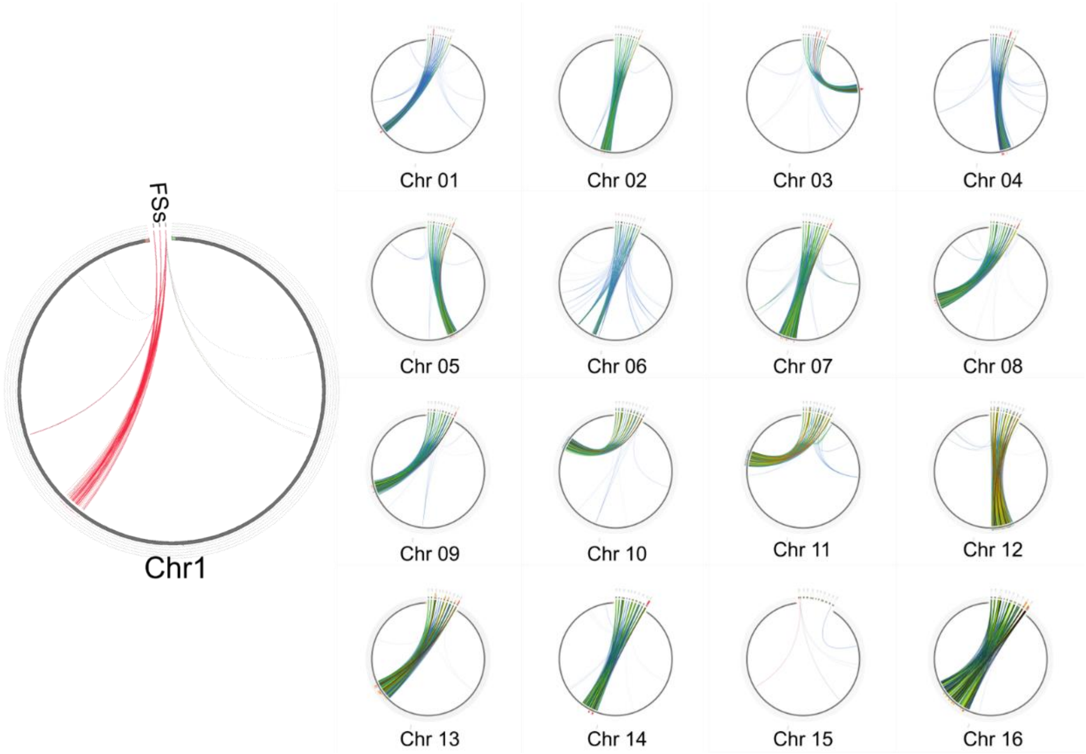

Fig. S3. Featured sequences (FSs) of centromere regions across the 16 chromosomes. The line colors indicate varying degrees of similarity. Blue, green, and orange indicate similarity values ranging from 0 to 50%, 50% to 75%, and 75% to 100%, respectively. Chromosome (Chl) 1 is shown as a representative example. All source data are provided as a Source Data file.

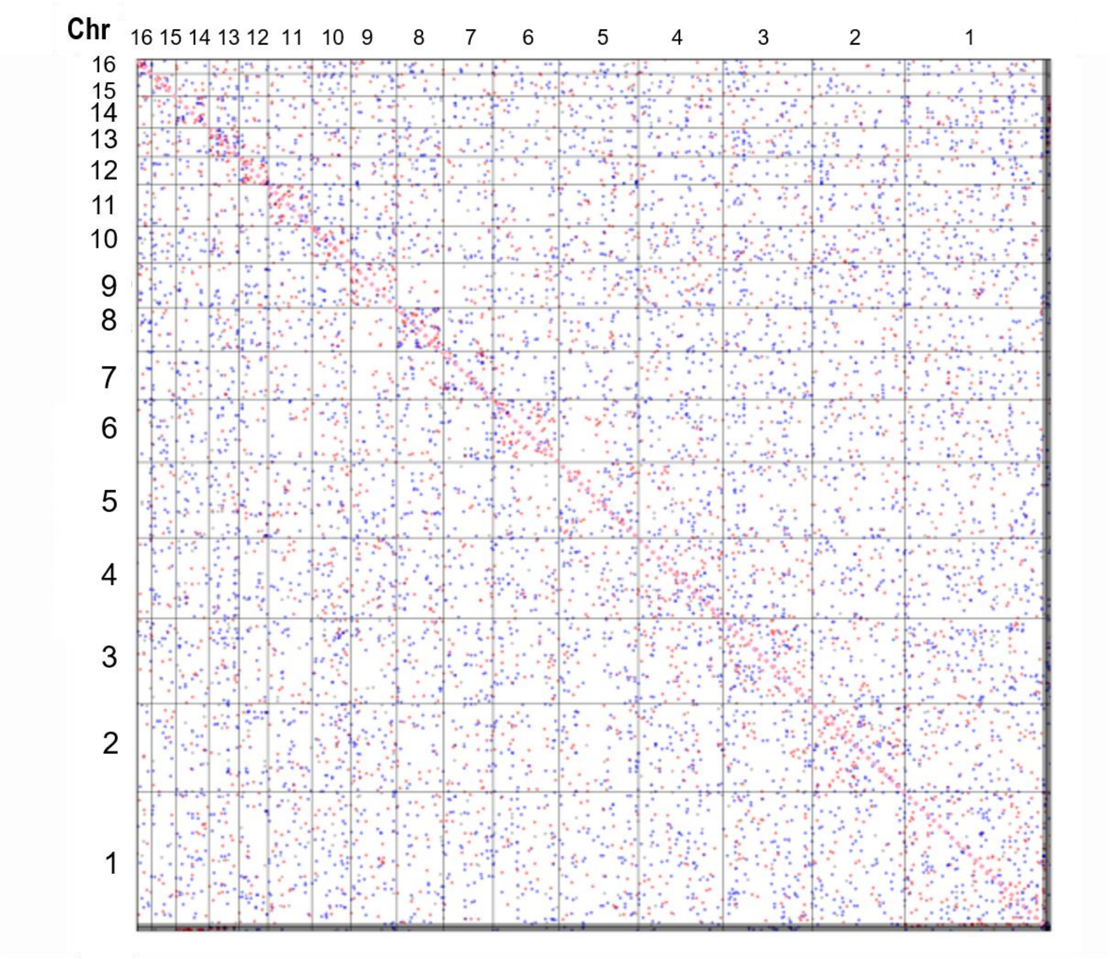

Fig. S4. Synteny analysis of the sixteen chromosomes of MEM25. Pairwise gene alignments were performed using BLAST. Genes exhibiting the highest sequence similarity are indicated by red dots, while blue dots represent genes among the top four homologous hits. Diagonal linear patterns reflect self-alignments, whereas off-diagonal linear arrangements suggest large-scale duplication events, including potential whole-genome duplications. All source data are provided as a Source Data file.

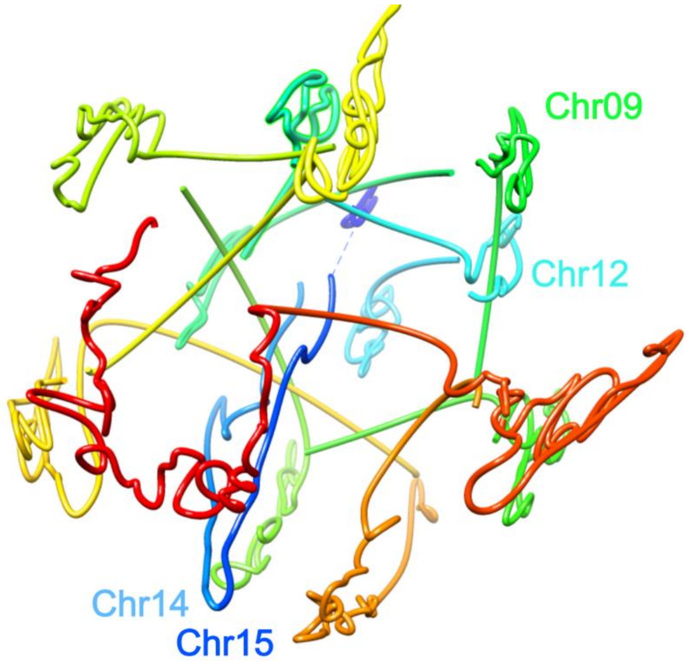

Fig. S5. Three-dimensional genome of MEM25 at a resolution of 100 kb. A relatively greater interaction is observed for two sets of chromosomes, Chr09 through Chr12 and Chr14 through Chr15, than the remaining ones. Chromosomes distinguished by various colors. All source data are provided as a Source Data file.

a

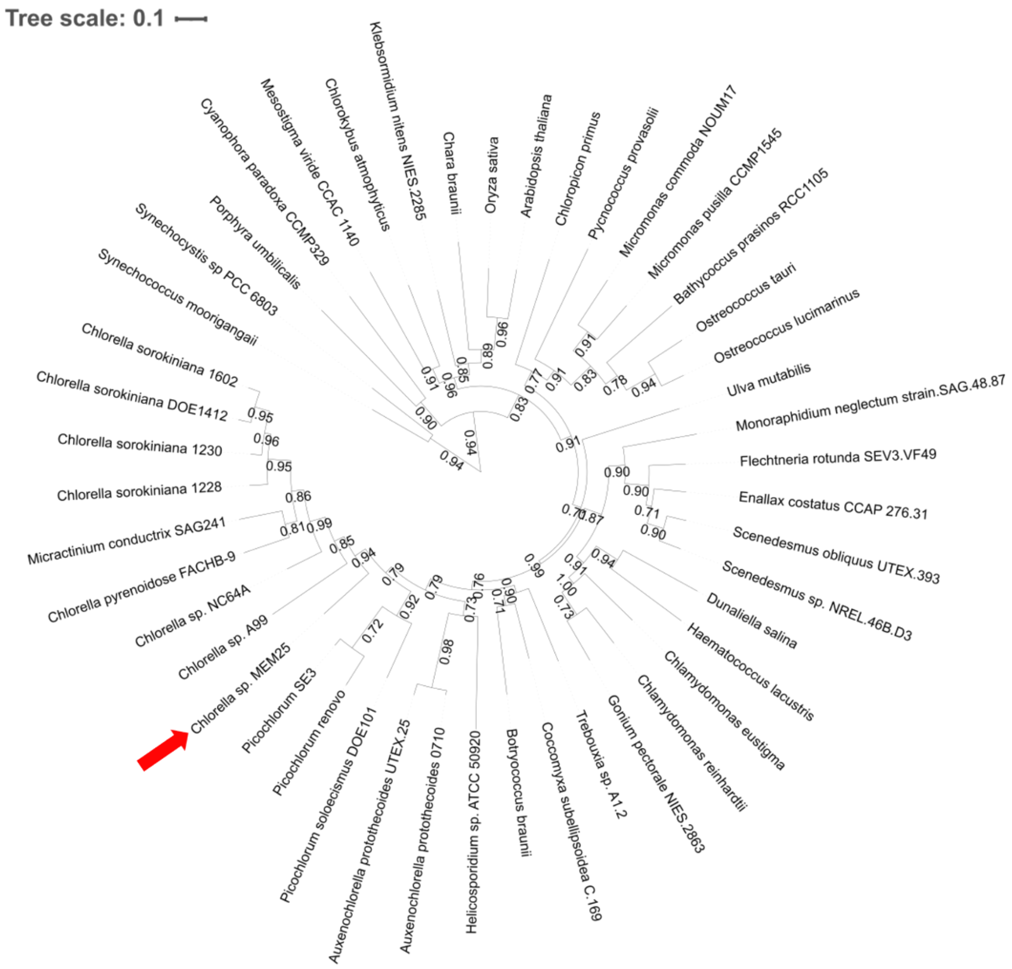
Tree scale: 0.1
Klebsormidium nitens NIES 2285
Chlorokybus atmophyticus
Mesostigma viride CCAC 1140
Cyanophora paradoxa CCMP329
Porphyra umbilicalis
Synechocystis sp PCC 6803
Synechococcus moorigangaii
Chlorella sorokiniana 1602
Chlorella sorokiniana DOE1412
Chlorella sorokiniana 1230
Chlorella sorokiniana 1228
Micractinium conductrix SAG241
Chlorella pyrenoidose FACHB-9
Chlorella sp. NC64A
Chlorella sp. A99
Chlorella sp. MEM25
Picochlorum SE3
Picochlorum renovo
Picochlorum soloecismus DOE101
Auxenochlorella protothecoides UTEX.25
Auxenochlorella protothecoides 0710
Helicosporidium sp. ATCC 50920
Botryococcus braunii
Coccomyxa subellipsoidea C.169
Trebouxia sp. A1.2
Gonium pectorale NIES.2863
Chlamydomonas reinhardtii
Chlamydomonas eustigma
Haematococcus lacustris
Dunaliella salina
Scenedesmus sp. NREL.46B.D3
Scenedesmus obliquus UTEX.393
Enallax costatus CCAP 276.31
Flechtneria rotunda SEV3.VF49
Monoraphidium neglectum strain.SAG.48.87
Ulva mutabilis
Ostreococcus lucimarinus
Ostreococcus tauri
Bathycoccus prasinos RCC1105
Micromonas pusilla CCMP1545
Micromonas commoda NOUM17
Pycnococcus provasolii
Chloropicon primus
Arabidopsis thaliana
Oryza sativa
Chara braunii

b

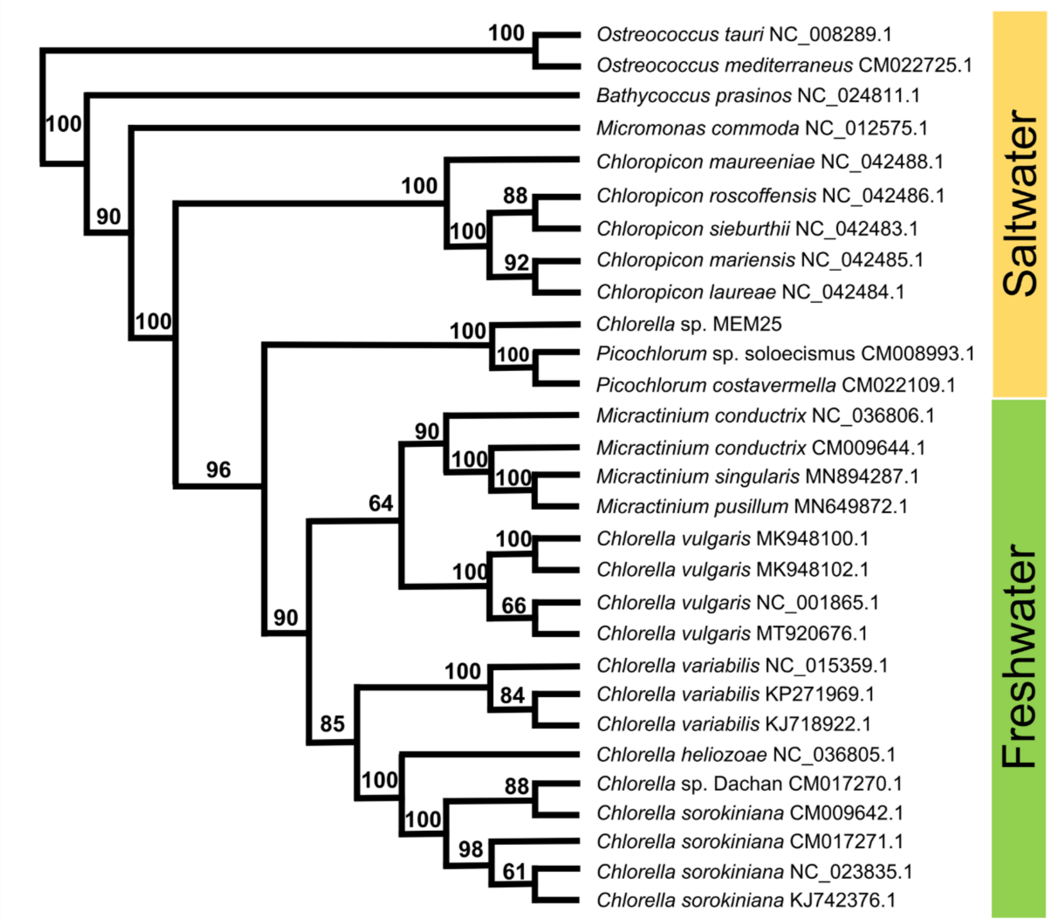

Fig. S6. Phylogenetic tree of selected species. (**a**) Phylogenetic tree of selected species with 1000 bootstrap replicates. (**b**) Chloroplast phylogenetic tree of selected species with available chloroplast genomes. All source data are provided as a Source Data file.

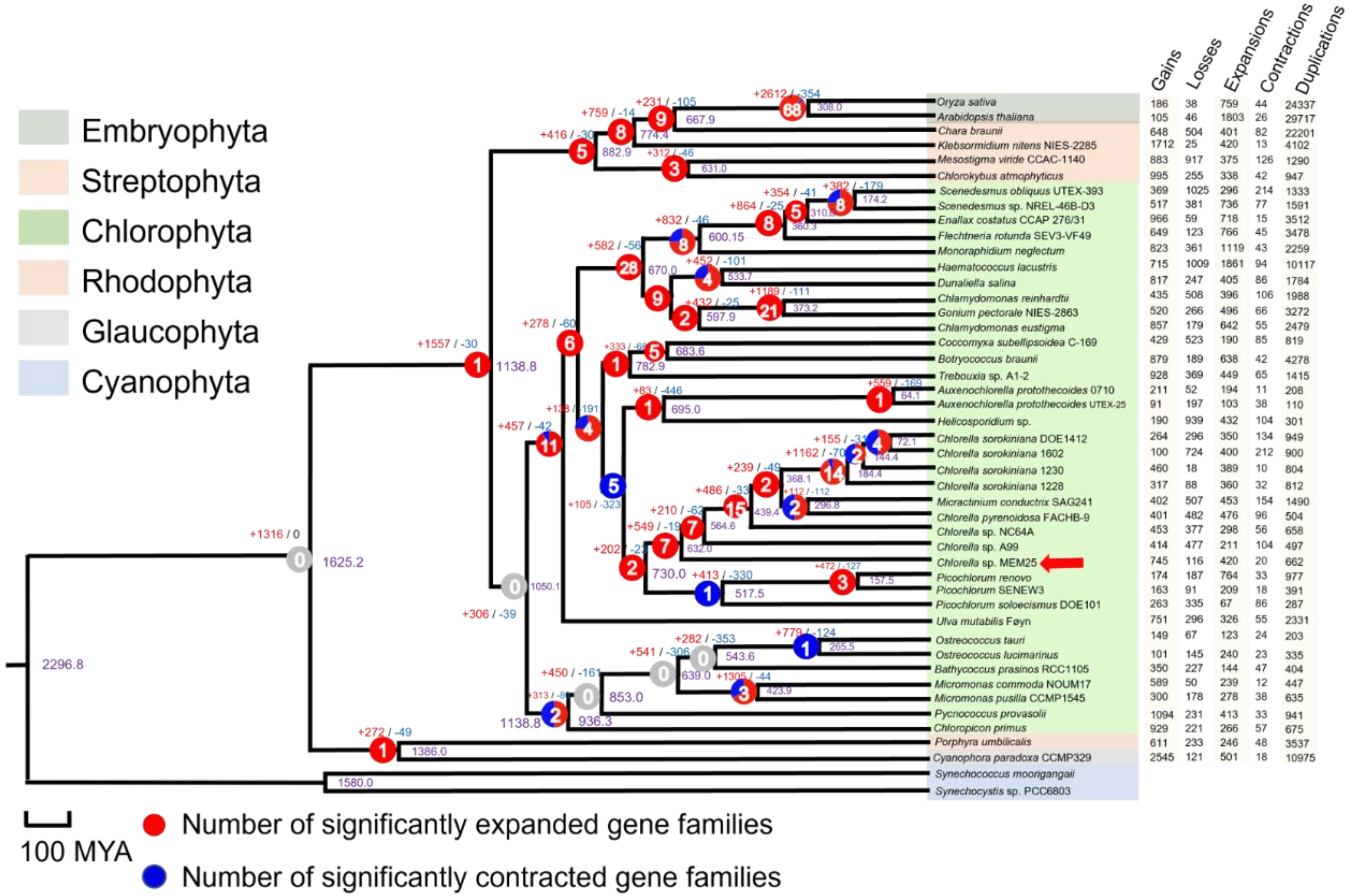
Embryophyta
Streptophyta
Chlorophyta
Rhodophyta
Glaucophyta
Cyanophyta
Gains
Losses
Expansions
Contractions
Duplications
Oryza sativa 186 38 759 44 24337
Arabidopsis thaliana 105 46 1803 26 29717
Chara braunii 648 504 401 82 22201
Klebsormidium nitens NIES-2285 1712 25 420 13 4102
Mesostigma viride CCAC-1140 883 917 375 126 1290
Chlorokybus atmophyticus 995 255 338 42 947
Scenedesmus obliquus UTEX-393 369 1025 296 214 1333
Scenedesmus sp. NREL-46B-D3 517 381 736 77 1591
Enallax costatus CCAP 276/31 966 59 718 15 3512
Flechtneria rotunda SEV3-VF49 649 123 766 45 3478
Monoraphidium neglectum 823 361 1119 43 2259
Haematococcus lacustris 715 1009 1861 94 10117
Dunaliella salina 817 247 405 86 1784
Chlamydomonas reinhardtii 435 508 396 106 1988
Gonium pectorale NIES-2863 520 266 496 66 3272
Chlamydomonas eustigma 857 179 642 55 2479
Coccomyxa subellipsoidea C-169 429 523 190 85 819
Botryococcus braunii 879 189 638 42 4278
Trebouxia sp. A1-2 928 369 449 65 1415
Auxenochlorella protothecoides 0710 211 52 194 11 208
Auxenochlorella protothecoides UTEX-25 91 197 103 38 110
Helicosporidium sp. 190 939 432 104 301
Chlorella sorokiniana DOE1412 264 296 350 134 949
Chlorella sorokiniana 1602 100 724 400 212 900
Chlorella sorokiniana 1230 460 18 389 10 804
Chlorella sorokiniana 1228 317 88 360 32 812
Micractinium conductrix SAG241 402 507 453 154 1490
Chlorella pyrenoidosa FACHB-9 401 482 476 96 504
Chlorella sp. NC64A 453 377 298 56 658
Chlorella sp. A99 414 477 211 104 497
Chlorella sp. MEM25 745 116 420 20 662
Picochlorum renovo 174 187 764 33 977
Picochlorum SENEW3 163 91 209 18 391
Picochlorum soloecismus DOE101 263 335 67 86 287
Ulva mutabilis Føyn 751 296 326 55 2331
Ostreococcus tauri 149 67 123 24 203
Ostreococcus lucimarinus 101 145 240 23 335
Bathycoccus prasinos RCC1105 350 227 144 47 404
Micromonas commoda NOUM17 589 50 239 12 447
Micromonas pusilla CCMP1545 300 178 278 38 635
Pycnococcus provasolii 1094 231 413 33 941
Chloropicon primus 929 221 266 57 675
Porphyra umbilicalis 611 233 246 48 3537
Cyanophora paradoxa CCMP329 2545 121 501 18 10975
Synechococcus moorigangaii
Synechocystis sp. PCC6803
2296.8
1625.2
+1316 / 0
1580.0
100 MYA
Number of significantly expanded gene families
Number of significantly contracted gene families

Fig. S7. Expansion and contraction of gene families in representative species of Viridiplantae. Branch length represents evolution time with average divergence times for each node indicated by purple numbers (million years, Ma). The red number with a plus sign (+) on each branch indicates expanded gene families, while the blue number with a minus sign (-) indicates the number of contracted gene families. Significantly expanded or contracted orthologs are denoted by filled red circles ● and filled blue circles●, respectively. The panel on the right displays the numbers of gains, losses, expansions, contractions, and duplications of orthologs in each species in comparison with the most recent common ancestor (MRCA) during recent differentiation. All source data are provided as a Source Data file.

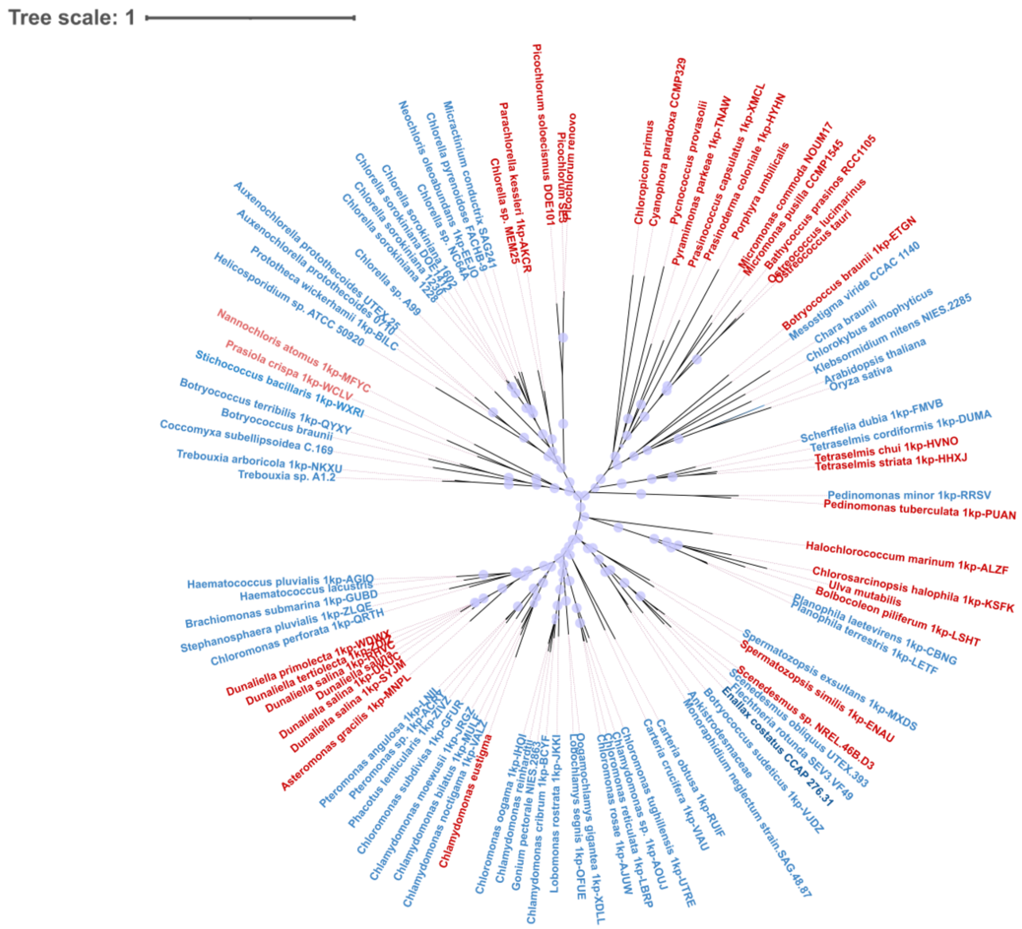

Fig. S8. Unrooted phylogenetic tree of Chlorophyta. Species with transcriptomic data from the 1KP project were included. Species with optimal biomass production in fresh water are marked in blue, while those adapted to seawater are marked in red. Purple dots denote branches with bootstrap support values exceeding 90% based on 1,000 replicates. Apparent displacement of the dots from branch points is due to overlapping branches. For detailed information on species from the 1KP project and their respective habitats, please refer to **Supplementary Data 6**. Note the sparse occurrence of some saltwater/fresheater species (e.g., *Dunalinna salina*, *Porphyra umbilicalis*), which is likely due to the lineage-specific and bidirectional nature of marine-freshwater transitions, as well as their specialization to unique niches. All source data are provided as a Source Data file.

a

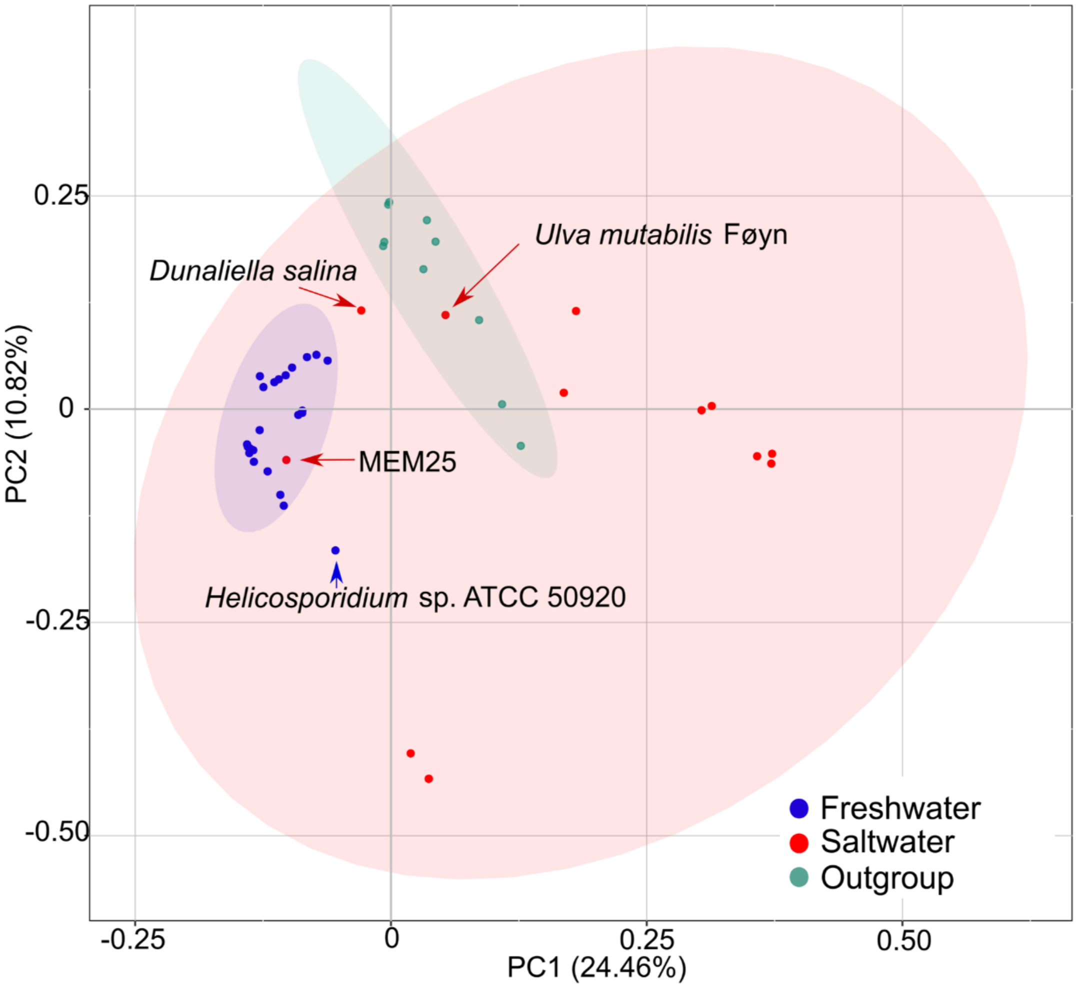

Ulva mutabilis Føyn
Dunaliella salina
MEM25
Helicosporidium sp. ATCC 50920
Freshwater
Saltwater
Outgroup
0.25
0
-0.25
-0.50
PC2 (10.82%)
-0.25
0
0.25
0.50
PC1 (24.46%)

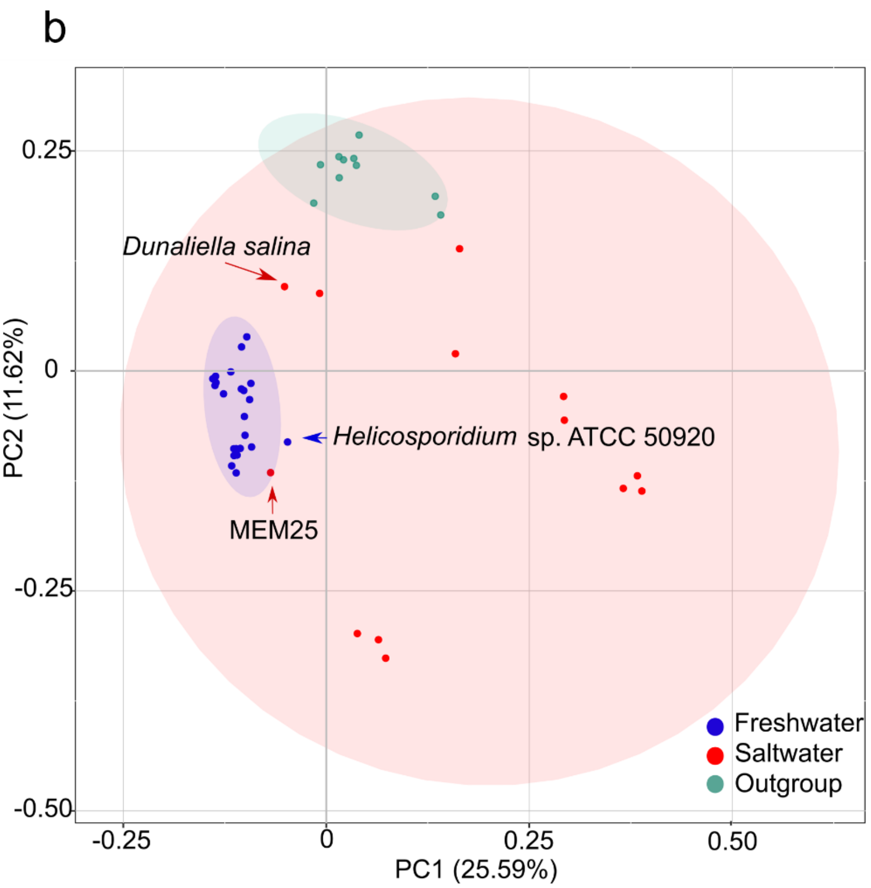

Fig. S9. Clustering of selected Chlorophyta species using a 60% cutoff (a) or an 80% cutoff (b). Principal Component Analysis (PCA) was conducted based on 614 or 311 featured gene families for either the 60% cutoff or the 80% cutoff. The corresponding ellipsoid signifies the 95% confidence interval for each cluster. Note that freshwater species formed a tight cluster away from the, more dispersed, saltwater species, with the exceptions of MEM25 and an obligate parasitic fresh-water Chlorophyta *Helicosporidium* sp. ATCC 5092. The latter stood distinct from all other freshwater species, possibly due to its adaptation to parasitic habitats [1], while MEM25 emerged as the only marine species within the freshwater species group. Pearson's chi-square (χ^2) tests confirmed the statistical significance ($P < 0.05$). Habitat-specific gene families were defined as follows: freshwater-specific families were those present in >80% of the 23 freshwater Chlorophyta species and in <40% of the 13 seawater species, with the inverse criteria applied for seawater-specific families. Using these thresholds, we identified 311 habitat-associated families (287 freshwater-specific, 24 seawater-specific). Pearson's chi-square test revealed a highly significant deviation from a uniform distribution ($\chi^2 = 108.72$, df = 1, Bonferroni-corrected $P = 6.84 \times 10^{-14}$). The effect size, Cramer's V = 0.59 (95% CI: [0.54, 0.64]), indicated a strong association between gene family composition and habitat type. All source data are provided as a Source Data file.

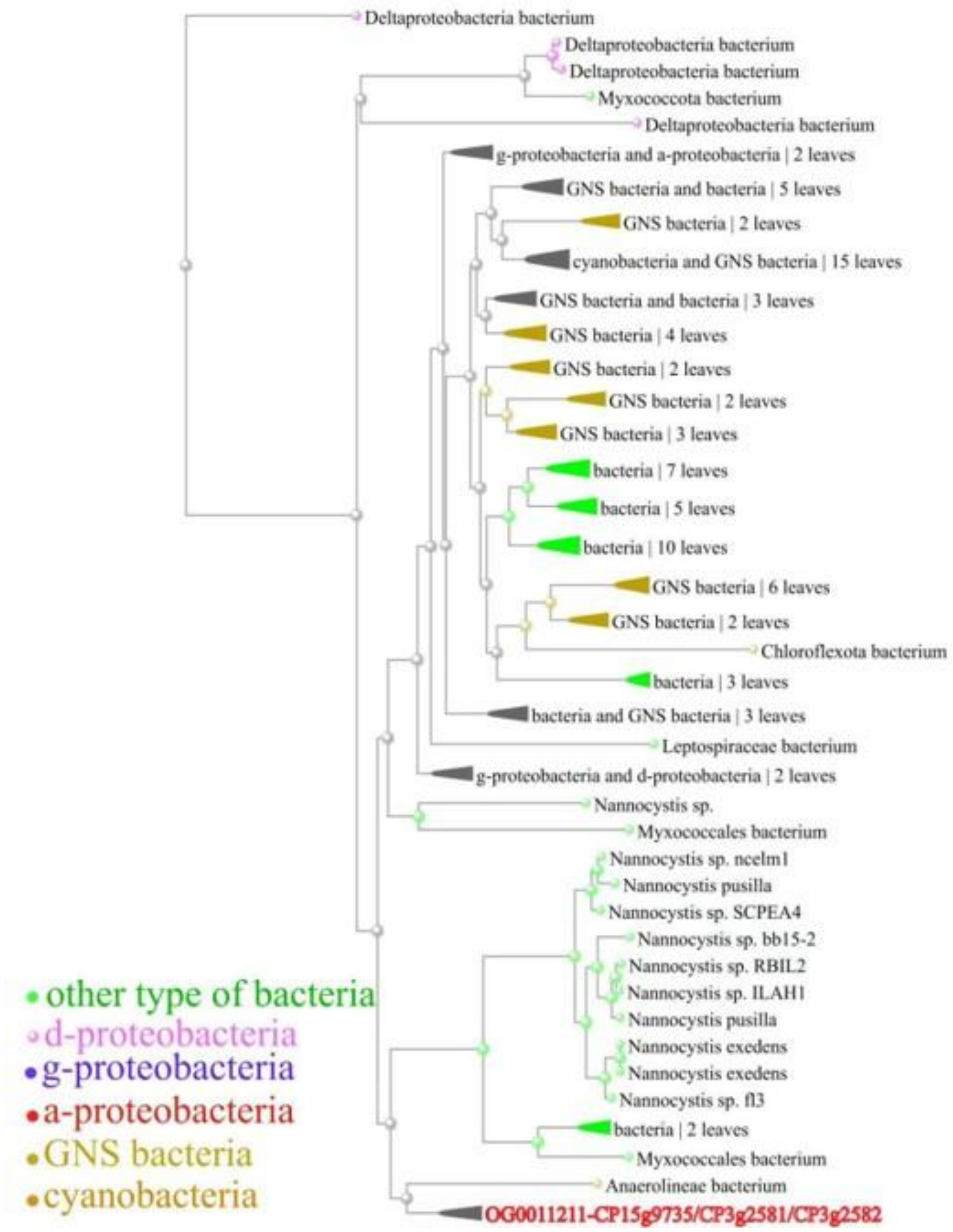

Fig. S10. MEM25 acquires OD0011211 from bacteria through gene transfer. All source data are provided as a Source Data file.

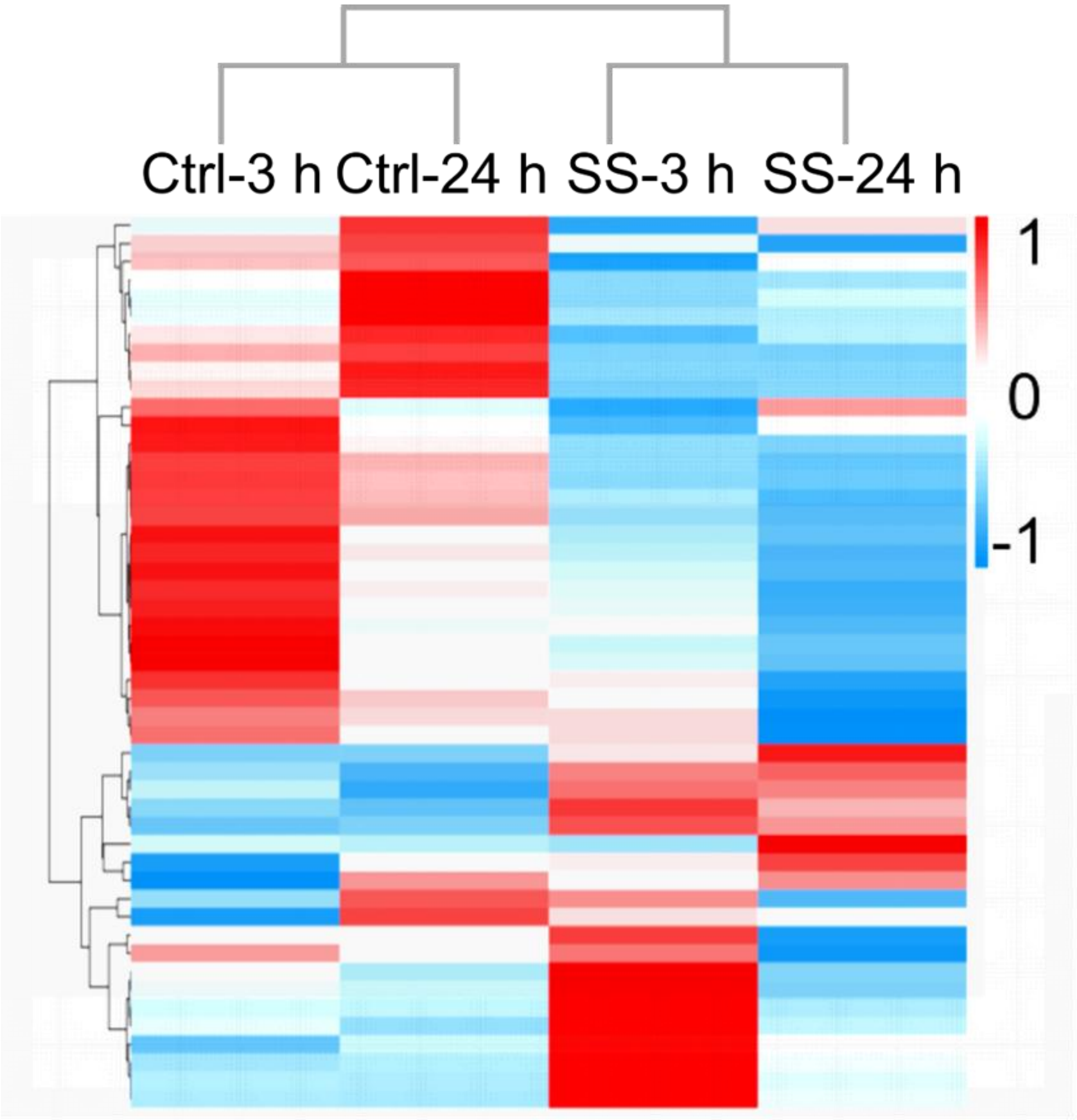

Fig. S11. Transcriptional dynamics of the 89 expanded orthogroups in *Chlorella* sp. MEM25 under the high-salinity condition. Ctrl, 35‰ salinity; SS, salt stress (105‰ salinity). The expression levels have been normalized using Z-score, where red represents high expression and blue represents low expression. All source data are provided as a Source Data file.

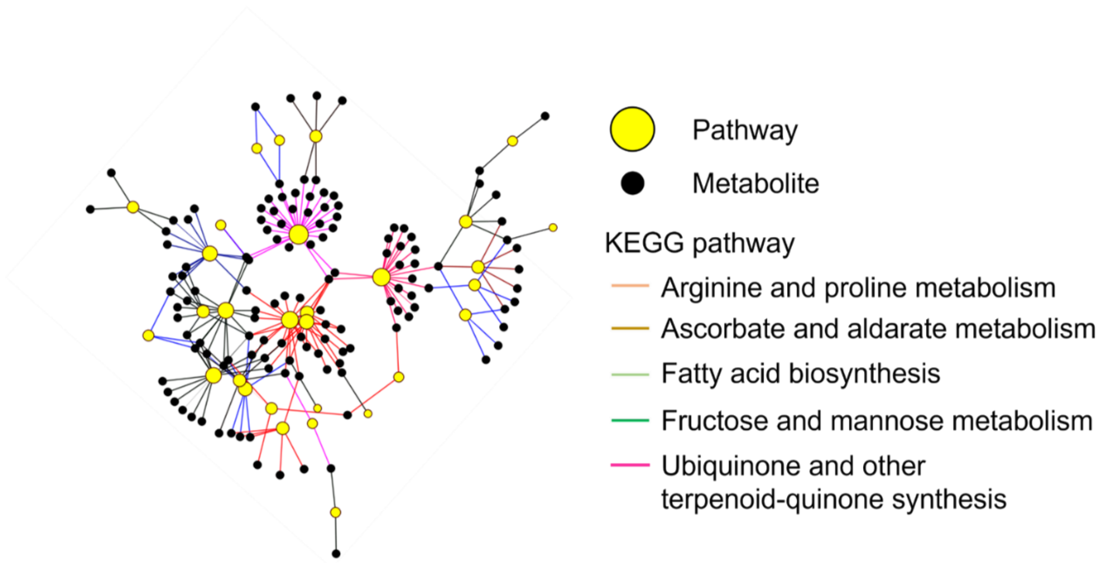

Fig. S12. Pathway enrichment analysis of differentially accumulated metabolites. Yellow bubbles represent pathways, while black bubbles denote metabolites. The size of the bubble corresponds to the number of metabolites or genes within each pathway. All source data are provided as a Source Data file.

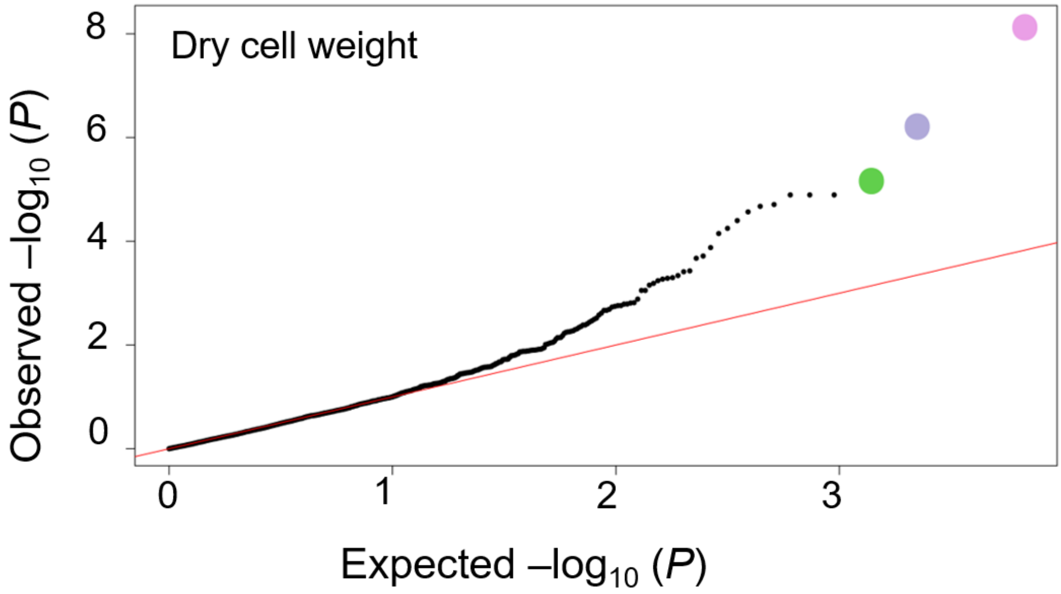

Fig. S13. Quantile-quantile (Q-Q) plot comparing observed versus expected *P* values of the GWAS results. The straight line on the Q-Q plot indicates the distribution of SNPs under the null hypothesis. Significant SNPs (FDR of 5%) are highlighted by non-black dots. All source data are provided as a Source Data file.

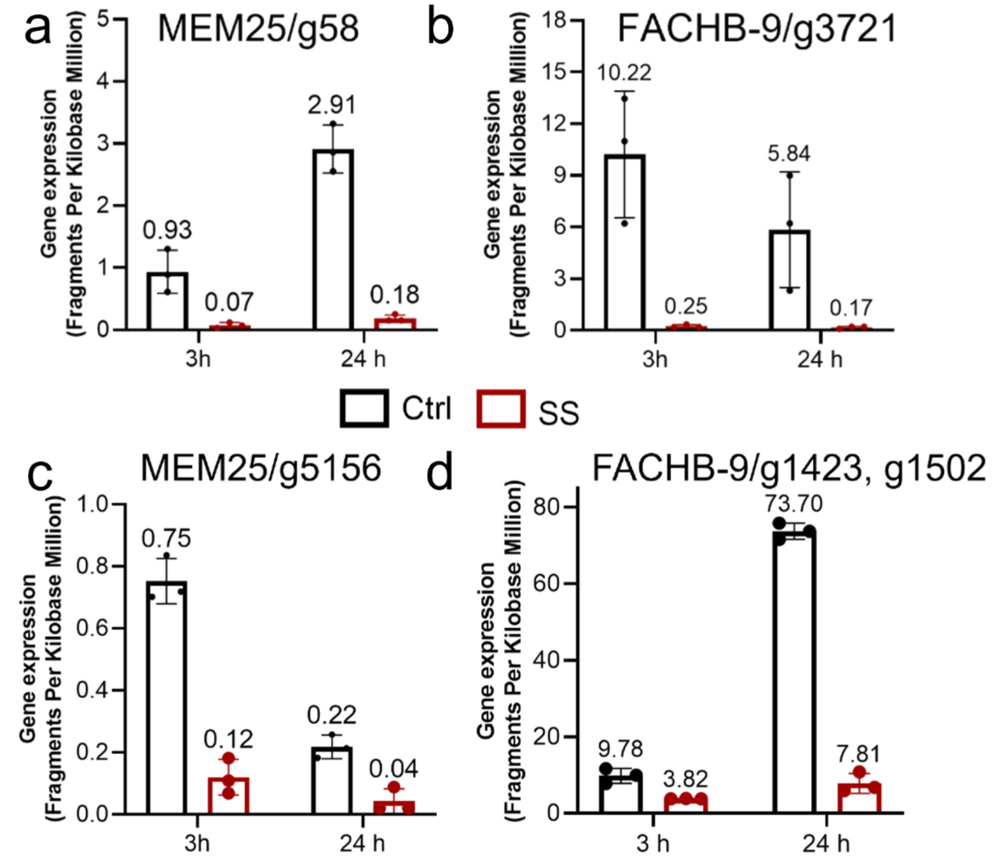

Fig. S14. Functional validation of newly discovered salinity-related genes. (a) Transcriptional dynamics of *CP1g58* in MEM25 under increased salinity. (b) Transcriptional dynamics of the gene *g3721* in freshwater FACHB-9 under increased salinity. (c) Transcriptional dynamics of *CP5g5156* in MEM25 under increased salinity. (d) Transcriptional dynamics of the genes *g1423* and *g1502* in freshwater FACHB-9 under increased salinity. Data are presented as the means ± SD (n = 3). Asterisks (*) indicate statistically significant differences ($P \leq 0.05$). All source data are provided as a Source Data file.

Supplementary Note 1

Highly contiguous genome assembly

We employed a hierarchical assembly approach, using 5.45 Gb (90.99-fold coverage) of PacBio long reads, 13.9 Gb (254.08-fold coverage) of Illumina short paired reads, and 9.24 Gb (185.38-fold coverage) of Hi-C data (**Table S1**). The integrated assembly yielded a high-quality nuclear genome for MEM25 of 53,506,137 bp, distributed over 16 chromosomes (**Fig. 1a**), as confirmed by 4’, 6-diamidino-2-phenylindole (DAPI) staining (**Fig. S1**). A single gap was present in the assembled genome at the end of chr15 (1,311,674 bp) due to the difficulty in assembling repetitive fragments of the genome. The scaffold N50 of the assembled sequence was 4,303,545 bp (longest, 4.3 Mb; **Supplementary Data1**), which was longer than previous records for microalgae [2-6] (**Table S2**), indicating a high level of genome scaffold continuity.

In the nuclear genome of MEM25, a total of 10,225 protein-coding genes were predicted, with 95.6% (9,775) being covered by mRNA-Seq data (defined as 80% of the transcribed region mapped by at least 10 reads) (**Table S3**). The genome assembly successfully captured 97.2% (with transcriptome mode; 93.7% with protein mode) of the Chlorophyta Benchmarking Universal Single Copy Orthologs (BUSCO) dataset, indicating a high level of gene region completeness in the genome assembly (**Table S4**). Various non-coding RNA sequences were identified and annotated in the MEM25 genome, including 97 transfer RNAs, 46 small nuclear RNAs, 31 micro RNAs, and 13 ribosomal RNAs (**Table S5**).

T2T assembled and centromere annotated chromosome sequences

Repetitive regions, especially centromeres and telomeres, are the most difficult regions to annotate. Notably, centromere coverage is lacking in Chlorophyta algal genomes. In the case of MEM25, the total repetitive and transposable element content

amounted to 5,164,600 bp in total (9.65%), including long terminal repeat elements (6.37%), unclassified repeats (1.38%), and long interspersed nuclear elements (0.8%) (**Table S6**). The assembled algal genome showed a high value of Long Terminal Repeat (LTR) Assembly Index (LAI) (15.33; a reliable metric validated in over 40 plant genomes [7]) (**Fig. S2**), meeting the quality benchmark for reference genomes (i.e., between 10 and 20) (**Supplementary Data2**) [7].

Additionally, the plant-specific telomeric repeats (TTTAGGG) were identified in all chromosome sequences (**Supplementary Data3**), with twelve of the sixteen chromosomes containing telomeric repeats at both ends, indicating the complete assembly of chromosome ends (**Fig. 1b**). Notably, all sixteen chromosomes were clearly marked with centromeres, with Chr15 identified as a telocentric chromosome (**Fig. 1b**). Three featured sequences (FSs) were identified with lengths of 154 bp, 152 bp, and 134 bp respectively (FSs in **Fig. S3**).

An investigation into intragenomic synteny among three *Chlorella* genomes (MEM25, *Chlorella pyrenoidosa* FACHB-9 [3], and *Chlorella sorokiniana* 1230 [8]), along with the genomes of *Chlamydomonas reinhardtii* and *Arabidopsis thaliana*, revealed no whole-genome duplications (WGDs) in the MEM25 genome. Despite the fragmented genome assembly of *C. pyrenoidosa* FACHB-9 (hereafter FACHB-9) and *C. sorokiniana* 1230, better synteny was observed between MEM25 and these strains than that between MEM25 and *C. reinhardtii* or *A. thaliana*, suggesting the sequence conservation of centromeres and telomeres in chlorella strains (**Fig. 1c**). The seeming synteny of MEM25 telomeres to one region in FACHB-9 genome is due to the fragmented assembly of the latter (1336 contigs). These results indicate that the quality, accuracy, and completeness of the MEM25 genome assembly is higher than the

available reference sequences [3,5], ensuring the reliability of subsequent genomic analyses.

A 3D, 100 kb resolution map of the MEM25 genome was constructed for chromosome conformation capture (**Fig. S4**) while whole-chromosomal interactions were assessed (**Fig. 1d**). Although two sets of chromosomes, Chr09 through Chr12 and Chr14 through Chr15 (**Fig. S4**), were associated more closely with each other than the remaining ones, more intense intra-chromosomal interactions (within the same chromosome) than inter-chromosomal interactions (between different chromosomes) were detected (Student's *t*-test, $P < 0.05$, **Fig. 1d**). This suggests that all components of the algal chromosomes occupy their own nuclear territories, which in turn facilitates intra-chromosomal interactions.

Table S1. Summary of *Chlorella* sp. MEM25 genome assembly by Illumina, PacBio, and Hi-C.

Assembly approaches	Parameters	Values
PacBio-based contigs	Total non-gap length(bp)	54,975,376
	Contig Number	53
	Contigs N50(bp)	3,763,867
Hi-C based pseudochromosomes	Total non-gap(bp)	53,622,205
	Contig Number	39
	Contigs N50(bp)	4,303,545
	Scaffold/pseudochromosome Number	22
	Scaffold/pseudochromosome N50(bp)	4,392,856
Illumina	Clean Reads	46,577,241
	Clean Bases	13,973,172,300
	Q30 Percent (read1)	94.82
	Q30 Percent (read2)	92.93

Table S2. Summary of sequenced genomes of *Chlorella* spp.

Species	Genome size (Mb)	Scaffold number	Gene number	Released year	References
Chlorella variabilis NC64A	46.16	414	9780	2010	PMID: 20852019
Chlorella sorokiniana BD09	53.97	920	9668	2019	PMID: 31267025
Chlorella sorokiniana BD08	58.69	1242	10240	2019	PMID: 31267025
Chlorella sp. Dachan	60.37	2277	9821	2019	PMID: 31267025
Chlorella sorokiniana 1602	59.57	159	10384	2018	DIO: 10.1016/j.algal.2018.09.012
Chlorella pyrenoidosa FACHB-9	56.993	1346	10577	2015	PMID: 26486592
Chlorella vulgaris UTEX259	39.13	780	9439	2019	PMID: 31794607
Chlorella sp. NJ7	39.08	753	9412	2019	PMID: 31794607
Chlorella sp. A99	40.93	82	8298	2018	PMID: 29848439

Table S3. Summary of gene structure prediction.

Method	The source of annotated results	Number	Average transcript length (bp)	Average CDS length (bp)
De novo	Augustus	7,110	4,390.08	1,561.65
	GlimmerHMM	39,758	1,149.28	839.05
	SNAP	10,712	4,704.25	1,170.91
	Geneid	16,113	2,430.71	1,087.66
	Genscan	6,654	7,206.67	2,632.82
Homolog	*Coccomyxa subellipsoidea* C-169	4,068	2,497.96	883.91
	Chlorella sorokiniana 1602	4,933	3,592.10	1,268.14
	Chlorella variabilis NC64A	4,339	2,240.18	812.82
	Auxenochlorella protothecoides	3,930	3,293.20	1,031.17
	Arabidopsis thaliana	3,051	2,532.32	924.4
	Chlamydomonas reinhardtii	4,308	2,184.85	805.44
	Dunaliella salina	3,745	2,096.47	751.07
	Micractinium conductrix SAG241	4,653	3,959.49	1,282.44
RNAseq	PASA	162,030	2,629.82	1,106.86
	Transcripts	23,421	7,535.32	4,020.96
EVM		10,743	3,564.55	1,428.09
Pasa-update*		10,623	3,594.93	1,465.58
Final set*		10,225	3,615.56	1,467.76

Note: *De novo*, which means using software to re predict genes; Homelog, which means to predict the gene of MEM25 using the Homeotic gene of the nearby species; RNAseq, which means to use the transcriptome data of MEM25 to predict new genes and transcripts; EvidenceModeler (EVM), means to integrate the above results; Pasa-update* , means to use of PASA software to update the EVM consensus predictions, adding UTR annotations and models for alternatively spliced isoforms; Final set*, means the final result after correcting and removing redundancy.

Table S4. Summary of Chlorophyta Benchmarking Universal Single.

**Copy Orthologs (BUSCO) assessment**	
Total gene number	10,225
Complete BUSCOs	1476 (97.20%)
Complete and single-copy BUSCOs	1459 (96.1%)
Complete and duplicated BUSCOs	17 (1.1%)
Fragmented BUSCOs	11 (0.7%)
Missing BUSCOs	32 (2.1%)
Total BUSCO groups searched	1519 (100%)

Note: Use chlorophyta database for BUSCO evaluation.

BUSCO version is: 4.0.6

The lineage dataset is: chlorophyta_odb10 (Creation date: 2019-11-20, number of species: 16, number of BUSCOs: 1519)

Summarized benchmarking in BUSCO notation for file CP.genome.rename.fa

BUSCO was run in mode: genome

Table S5. Summary of non-coding RNAs.

Type	Copy number	Average length(bp)	Total length(bp)	% of genome
miRNA	31	120.81	3,745	0.0070
tRNA	97	76.7	9,894	0.0185
rRNA	13	300.67	6,314	0.0118
18S	3	554.78	4,993	0.0093
28S	5	110.22	992	0.0019
5.8S	3	109.67	329	0.0006
5S	2	0	0	0.0000
snRNA	46	126.15	5,803	0.0108
scaRNA	1	125	125	0.0002
Unknown	1	273	273	0.0005

Note: MicroRNA (miRNA); Transfer RNA (tRNA); Ribosomal RNA (rRNA); 18S, 28S, 5.8S and 5S, four Types of rRNAs in Eukaryote; Small nuclearRNA (snRNA); Small Cajal body-specific RNA (scaRNA).

Table S6. Summary of repetitive and transposable elements.

Type	*De novo*+Repbase		TE Proteins		Combined TEs	
	Length (bp)	% in Genome	Length (bp)	% in Genome	Length (bp)	% in Genome
**DNA**	337,080	0.63	2,145	0	338,211	0.63
**LINE**	427,770	0.8	5,989	0.01	429,433	0.8
**SINE**	198	0	0	0	198	0
**LTR**	3,416,951	6.37	600,016	1.12	3,439,312	6.41
**Unknown**	740,411	1.38	0	0	740,411	1.38
**Tandem repeat**	277,753	0.52	0	0	277,753	
**Total**	5,141,787	9.6	608,150	1.13	5,164,600	9.65

Note: Tandem repeat (TE); *De novo*+Repbase, means conducting ab initio prediction and retrieving from the repeat sequence database (REPBASE); TE Proteins, means that Transposable element elements (TE proteins) are obtained by annotating genomes based on RepBase library through RepeatProteinMask software; Combined TEs, means that the result of integration.

Note: DNA, DNA Transposable element and Retrotransposon of DNA-DNA transposition; LINE, long interspersed nuclear elements; SINE, shortinterspersed nuclear element; LTR, Long terminal repeat-retrotransposons.

References

1 Pombert, J. F., Blouin, N. A., Lane, C., Boucias, D. & Keeling, P. J. A lack of parasitic reduction in the obligate parasitic green alga *Helicosporidium*. *PLoS Genetics* **10**, e1004355 (2014).

2 Cecchin, M. *et al. Chlorella vulgaris* genome assembly and annotation reveals the molecular basis for metabolic acclimation to high light conditions. *The Plant Journal* **100**, 1289-1305 (2019).

3 Fan, J. *et al.* Genomic foundation of starch-to-lipid switch in oleaginous *Chlorella* spp. *Plant Physiology* **169**, 2444-2461 (2015).

4 Goemann, C. L. C. *et al.* Genome sequence, phylogenetic analysis, and structure-based annotation reveal metabolic potential of *Chlorella* sp. SLA-04. *Algal Research* **69**, 102943(2023).

5 Blanc, G. *et al.* The *Chlorella variabilis* NC64A genome reveals adaptation to photosymbiosis, coevolution with viruses, and cryptic sex *The Plant Cell* **22**, 2943-2955 (2010).

6 Wang, Y. *et al.* Early stage adaptation of a mesophilic green alga to Antarctica: Systematic increases in abundance of enzymes and LEA proteins. *Molecular Biology and Evolution* **37**, 849-863 (2020).

7 Ou, S., Chen, J. & Jiang, N. Assessing genome assembly quality using the LTR Assembly Index (LAI). *Nucleic Acids Research* **46**, e126-e126 (2018).

8 Hovde, B. T. *et al.* Genomic characterization reveals significant divergence within *Chlorella sorokiniana* (Chlorellales, Trebouxiophyceae). *Algal Research* **35**, 449-461 (2018).
