# Supplementary material for: Cross-species dissection of saline-related genes by genetically deciphering a euryhaline microalga Chlorella sp
Source: Nat Commun. 2026 Jan 13;17:1577. doi: 10.1038/s41467-026-68287-6 (PMC12902096; doi:10.1038/s41467-026-68287-6)
Supplement: Supplementary file 2 — Descriptions of Additional Supplementary Files [file 41467_2026_68287_MOESM2_ESM.pdf]

Descriptions of Additional Supplementary Files

File Name: Supplementary Data 1

Description: Summary statistics for chromosome-scale assembly.

File Name: Supplementary Data 2

Description: Comparison of LAI values and assembly quality levels.

File Name: Supplementary Data 3

Description: The primary DNA tandem repeat sequence identified within the telomere structures.

File Name: Supplementary Data 4

Description: The 36 Chlorophyta species used for genome comparison analysis.

File Name: Supplementary Data 5

Description: Chlorophyta-conserved single-copy orthogroups identified across 36 species of chlorophyta.

File Name: Supplementary Data 6

Description: Species from the 1KP project and their respective habitats. Note: Unless specified otherwise, the habitats of individual species are typically indicated on the AlgaeBase website (https://www.algaebase.org/).

File Name: Supplementary Data 7

Description: Featured gene families enriched in either saltwater or freshwater Chlorophyta species identified through a machine learning strategy.

File Name: Supplementary Data 8

Description: Featured gene families enriched in either saltwater or freshwater Chlorophyta species derived by the cutoff method (60% cutoff).

File Name: Supplementary Data 9

Description: Statistics details of module-trait relationships.

File Name: Supplementary Data 10

Description: Alignment ratio of MEM25 and FACHB-9 transcriptomic reads on reference genome sequences.

File Name: Supplementary Data 11

Description: Parameters used to calculate the associations of each module. Note: The parameters include stress severity (i.e., salinity), stress duration (i.e., treatment duration), damaged state (the value is 0 if the alga is under the favored salinity, or 1 if not), and MEM25-specific mechanism (the value is 0 if the mechanism is specific to FACHB-9, or 1 if specific to MEM25).

File Name: Supplementary Data 12

Description: Genes in thistle2 module that are specifically associated with the saline adaptation of MEM25.

File Name: Supplementary Data 13

Description: Metabolic module significantly and specifically associated with the saline adaptation of MEM25 (the black and pink modules).

File Name: Supplementary Data 14

Description: Statistical details Gains and expansions of saline-related genes within the Viridiplantae.

File Name: Supplementary Data 15

Description: Differential expression analysis of genes undergone contraction or expansion.

File Name: Supplementary Data 16

Description: Values of Fragments Per Kilobase of exon model per Million mapped fragments for the enriched genes that undergone expansion.

File Name: Supplementary Data 17

Description: Transcriptional dynamics of the genes involved in proline biosynthesis and fatty acid desaturation.

File Name: Supplementary Data 18

Description: The dynamics of the 150 metabolites that have exhibited significant changes under high salinity (70‰ salinity).

File Name: Supplementary Data 19

Description: Summary of genes used for functional validation.

File Name: Supplementary Data 20

Description: Primers used in this study.
