# Supplementary material for: Cross-species dissection of saline-related genes by genetically deciphering a euryhaline microalga Chlorella sp
Source: Nat Commun. 2026 Jan 13;17:1577. doi: 10.1038/s41467-026-68287-6 (PMC12902096; doi:10.1038/s41467-026-68287-6)
Supplement: Supplementary file 4 — Reporting Summary [file 41467_2026_68287_MOESM4_ESM.pdf]

nature portfolio

Corresponding author(s): Yandu LU

Last updated by author(s): Nov 25, 2025

Reporting Summary

Nature Portfolio wishes to improve the reproducibility of the work that we publish. This form provides structure for consistency and transparency in reporting. For further information on Nature Portfolio policies, see our Editorial Policies and the Editorial Policy Checklist.

Statistics

For all statistical analyses, confirm that the following items are present in the figure legend, table legend, main text, or Methods section.

n/a	Confirmed	
☐	☐	The exact sample size (n) for each experimental group/condition, given as a discrete number and unit of measurement
☐	☒	A statement on whether measurements were taken from distinct samples or whether the same sample was measured repeatedly
☐	☐	The statistical test(s) used AND whether they are one- or two-sided<br>*Only common tests should be described solely by name; describe more complex techniques in the Methods section.*
☐	☐	A description of all covariates tested
☐	☒	A description of any assumptions or corrections, such as tests of normality and adjustment for multiple comparisons
☐	☒	A full description of the statistical parameters including central tendency (e.g. means) or other basic estimates (e.g. regression coefficient) AND variation (e.g. standard deviation) or associated estimates of uncertainty (e.g. confidence intervals)
☐	☒	For null hypothesis testing, the test statistic (e.g. F, t, r) with confidence intervals, effect sizes, degrees of freedom and P value noted<br>*Give P values as exact values whenever suitable.*
☐	☒	For Bayesian analysis, information on the choice of priors and Markov chain Monte Carlo settings
☐	☒	For hierarchical and complex designs, identification of the appropriate level for tests and full reporting of outcomes
☐	☐	Estimates of effect sizes (e.g. Cohen's d, Pearson's r), indicating how they were calculated

Our web collection on statistics for biologists contains articles on many of the points above.

Software and code

Policy information about availability of computer code

Data collection	All data supporting the findings of this study are publicly available. The Whole Genome Shotgun project for MEM25 has been deposited at NCBI GenBank under the accession JBSQDC000000000 [https://www.ncbi.nlm.nih.gov/nuccore/JBSQDC000000000.1], which includes the complete genome sequence. Raw sequencing data are available under NCBI BioProject accessions PRJNA1344311 [https://www.ncbi.nlm.nih.gov/bioproject/PRJNA1344311/] (sequencing data for assembly, including Illumina, Hi-C, and PacBio data) and PRJNA1344302 [https://www.ncbi.nlm.nih.gov/bioproject/PRJNA1344302/] (GWAS mutant resequencing data comprising 198 samples). The RNA-seq data have been deposited under the NCBI BioProject accession PRJNA1370502 [http://ncbi.nlm.nih.gov/bioproject/PRJNA1370502/], including raw transcriptome sequencing data from a total of 24 samples of MEM25 and FACHB-9. The metabolomics data are deposited in the EMBL-EBI MetaboLights database under accession MTBLS13425 [https://www.ebi.ac.uk/metabolights/editor/MTBLS13425], which encompasses raw metabolomics sequencing data, processed metabolite contents, and corresponding annotations derived from a total of 12 samples of MEM25 and FACHB-9. The sequences included in the comparative genomic and phylogenetic analysis of this study have been made available via FigShare with DOI 10.6084/m9.figshare.30811226 [https://doi.org/10.6084/m9.figshare.30811226]. Additionally, the integrated multi-omics data of MEM25 used in this study have been deposited in Figshare with DOI 10.6084/m9.figshare.29062721 [https://doi.org/10.6084/m9.figshare.29062721]. Source are provided with this paper.
Data analysis	Genome assembly<br>Single molecule real time sequencing (SMRT) libraries and Illumina short read libraries were constructed using fresh mid-log phase algae (BioMarker, China), followed by sequencing with the PacBio Sequel II and HiSeq 2000 platforms. The Hi-C library and sequencing were conducted with the Illumina HiSeq platform (Novogene, China). Multiple approaches have been employed to guarantee the quality of genome sequences. PacBio and Hi-C data, Hifiasm (version 0.16.1-r375) was used for subgenome splitting. SMRT sequencing data were assembled using Falcon (version 0.3.0) and CANU (version 2.1.1). 3D-DNA (version 180922) and ALLHiC (version 0.9.8) were used for draft genome correction and genome assembly, respectively. Finally, Juicebox (version 1.11.08) and Pilon (version 1.24) were used to correct genome

fragments (based on the chromosome contact frequency matrix) and base errors of the genome with Illumina and RNA-seq data, respectively. The Hi-C interaction frequency matrix was analyzed by Homer, FitHic (version 2.0.8), HiCExplorer (version 3.7.2), and HiCCUPS (version 5.14). The 3-D chromosome coordinate location was calculated using HiC-GNN and was visualized using UCSF Chimera (version 1.15).
Genome annotation and analyses
For genome annotation, various references were used, including A. thaliana, Auxenochlorella protothecoides UTEX25, Chlamydomonas reinhardtii, Chlorella sorokiniana 1602, Chlorella variabilis NC64A, Coccomyxa subellipsoidea C-169, Dunaliella salina CCAP1918, and Micractinium conductrix SAG241. Augustus (version 3.3.3), GlimmerHMM (version 3.0.4), SNAP (version 2013.11.29), Geneid (version 1.4), and Genscan were used to predict gene structure ab initio. Cufflinks (version 2.2.1) was used to predict novel genes and correct gene structure. EVidenceModeler (version 1.1.1) and PASA (version 2.4.1) were used to remove redundancy and correct the integrated annotation results.
Gene function was inferred using BLASTP (with a cutoff of e-value < 1×10^{-5}) by comparing with known protein sequence databases, such as Evolutionary genealogy of genes: Non-supervised Orthologous Groups (eggNOG), National Center for Bio-technology Information (NCBI), International Protein Resource Information System (InterPro)40, The Protein Families Database (Pfam), SwissPro, and KEGG.
Conserved non-coding DNA sequence annotation
The repeat sequences were annotated using RepeatMasker (version 4.1.0) while conserved non-coding DNA sequences (CNSs) were identified using dCNS (version 0.4). For CNSs annotation, the reference genomes included those of Chlorella sp. A99, A. thaliana, A. protothecoides UTEX-25, C. reinhardtii, D. salina, C. sorokiniana 1230, C. pyrenoidosa FACHB-9, M. conductrix SAG241, C. variabilis NC64A, Ostreococcus tauri, and Picochlorum soloecismus DOE101. The short segment tandem repeat sequences were eliminated and the adjacent CNSs with regional overlap or with spans less than 50 bp were merged. Finally, a total of 721 CNSs was obtained.
Telomere and centromere scanning
Tandem repeats finder (TRF, version 4.09) was used to search for tandem repeat sequences and identify telomere sequences. For centromere annotation, minimap2 (version 2.2.26) was used to mask the functional regions, followed by removal of redundancy and searching and extraction of the repeated units. The genome sequences were segmented into fragments with a length of 500 bp. CD-hit (version 4.8.1) was used to find the sequences with similarities above 70%. MEME SUITE (version 5.3.0) and Homer were used to extract the featured fragments of these sequences, which were subsequently aligned to the individual chromosome. The sequence similarity was visualized by Circoletto.
Genome quality assessment and collinearity analysis
LTR_FINDER (version 1.07) was used to identify Long terminal repeat retrotransposons (LTR-RT), this comprises a non-redundant LTR-RT library, with LTR Assembly Index (LAI) values calculated. Gene annotation and assembly were evaluated via BUSCO (version 5.2.2) using the single copy Homeotic gene database of Chlorophyta (https://busco-data.ezlab.org/v5/data/lineages/chlorophyta_odb10.2020-08-05.tar.gz). The genome synteny and collinearity were detected by JCVI (version 1.3.5) and the results were visualized by the R package Rideogram (version 0.2.2).
Comparative genomic and phylogenetic analysis
Genome sequences for the selected species were retrieved from the NCBI (https://www.ncbi.nlm.nih.gov/) and IMG-M databases (https://img.jgi.doe.gov/cgi-bin/m/main.cgi) (Supplementary Data4). OrthoFinder2 (version 2.5.2) was used for orthogroup inference, with 39,321 orthogroups obtained for the 46 selected species. Among them, 199 orthogroups were present in all the selected species, and these were used to infer species trees by using the STAG algorithm. Dollo parsimony was implemented for Count to infer family- and lineage-specific characteristics across the evolutionary tree.
Chloroplast genome sequences were retrieved from the CGIR database (https://ngdc.cncb.ac.cn/cgir/). Parsed hits for all species were aligned using MAFFT7 (version 7.480). Gaps and ambiguously aligned sites were removed using gBlock (version 0.91b). Sequences that caused aberrant alignments and whose real identity could not be confirmed were removed manually. Phylogenetic analyses were performed with a maximum likelihood method using IQ-TREE (version 2.1.4-beta).
The R8S script was employed to estimate temporal divergence based on the molecular evolution rate and stable fossil nodes. A strict clock model was used to avoid horizontal gene transfer and other events that affected divergence times. Multiple time constraints (fossil records for no less than three species within the same genus) were incorporated to evaluate our results using fossil cross-validation. The fossil records used in this study are relevant ones that have previously been applied to estimate the divergence times of eukaryotes, including those for (1) A. thaliana to Oryza sativa (115-308 Mya), (2) Mesostigma viride to Chlorokybus atmophyticus (174-631 Mya), (3) Ostreococcus lucimarinus to Micromonas pusilla (333-639 Mya), (4) Synechocystis sp. PCC 6803 to Synechococcus moorigangaii (1580-2557 Mya), and (5) Porphyra umbilicalis to Cyanophora paradoxa CCMP329 (1386-1680 Mya). The fossil records are available at Timetree (http://www.timetree.org/).
Genome-wide association
A mutant pool for MEM25 was generated by EMS mutagenesis following our previous report. In brief, the mutagenesis conditions were optimized by treating log-phase cells with different concentrations of EMS for varying durations, followed by assessing the number and morphology of the resulting colonies. A mutant pool was constructed using the optimized conditions and candidate mutants were further assessed for growth in either the low salinity (i.e., 35‰) or the high salinity (i.e., 70‰) conditions. The turbidity was monitored by recording OD750 at indicated intervals. A total of 536 mutants with altered OD750 were selected for further phenotyping under the high-salinity conditions. The 365 mutants with stable phenotypes after ten generations were assessed further for growth in the high salinity conditions (i.e., 70‰). Dry weight, cell size, and cell number were recorded. Finally, genomes of 195 salinity-related mutants and three WT samples were resequenced using the second-generation sequencing Illumina platform for PE150 sequencing (Novogene Company, China).
Fastp (version 0.20.0) was used for filtration and quality control of original sequencing data (fastp -t 10 -f 10 -F 10 -T 10 -W6 -u 20 -n 10 -c). Genome alignment was conducted using BWA-MEME (version 0.7.17-r1188) and SAMtools (version 1.12) (bwa mem -R '@RG\tID:{sample}\tSM:{sample}\tPL:Illumina'\ index fq1 fq2 | samtools sort -@ 2 -m 1G | samtools view -h -b -q30 > q30.sort.bam), followed by quality control using Qualimap (version 2.2.2). PCR duplicates and optical duplicates were removed using Picard's MarkDuplicates program (https://broadinstitute.github.io/picard/). Only high-quality sequencing data were used for the subsequent analysis. Variations were detected using Genome Analysis Toolkit (GATK) (version 4.2.1). A value of 0.00743 was obtained for the genomic heterozygosity of MEM25 by using a combination of Jellyfish (version 2.3.0) and GenomeScope (version 2.0). Genome alignment on the sequences around INDEL (Insertion and Deletion) was performed to detect variations for each sample. The results of all samples were merged when population variations, SNPs, and INDEL were obtained. Quality control on SNPs (QUAL < 30 || MQ < 40.00 || SOR > 4.000 || QD < 2.00 || FS > 60.000 || MQRankSum < -10.000 || ReadPosRankSum < -10.000 || ReadPosRankSum > 10.000) and INDEL (QUAL < 30 || MQ < 40.00 || SOR > 10.000 || QD < 2.00 || FS > 200.000 || ReadPosRankSum < -20.000 || ReadPosRankSum > 20.000) was conducted separately. Variations with abnormal sequencing depth were removed. GWAS analysis was performed using TASSEL, employing a Mixed linear model and Bonferroni correction. Variations were annotated via SnpEff. R package CMplot (https://github.com/g-insana/CMPlot.jl) was used to draw a Manhattan plot, Quality-Quality plot, and genome variation distribution maps.
Sample preparation for transcriptome and metabolome analysis
Cells were cultured into log phase under optimal conditions. Salt stress was induced by transferring microalgae to high-salinity conditions (that is 35 $g \cdot L^{-1}$ for FACHB-9 and 105 $g \cdot L^{-1}$ for MEM25). Aliquots of cells were collected following the salinity shifts for either transcriptome (after 3 h and 24 h) or metabolome (after 24 h) analysis. Triplicates were used for each treatment.
Transcriptome analysis

Total RNA extraction, sequencing, and assembly were performed according to previous reports 38. Transcriptome sequencing was completed by BioMarker Company (China, Beijing). Quality control and removal of low-quality fragments was conducted by FastQC (version 53.0) and fastp, followed by the trimming of low-quality regions via Trimmomatic (version 0.39). The RNAseq data were compared with the reference genomes using GSNAP (version 2021-07-23), followed by quantification of the values of FPKM (Fragments Per Kilobase of exon model per Million mapped fragments) using StringTie (version 2.1.7), R package Ballgown (version 2.22.0), and DESeq2. Differentially expressed genes were defined as those with FDR-adjusted p-value ≤ 0.05 and |log2FoldChange| ≥ 1. Function and pathway enrichment analysis was conducted using KEGG (https://www.kegg.jp/) and the R package clusterProfiler (version 3.18.1).
Metabolome analysis
Approximately 500 mg of algal biomass was collected, followed by metabolite extraction. Algal cells were suspended in 1 mL mixture of methanol and water (7/3, vol/vol) and kept at −80 °C for 2 min. The internal standard used was 2-chloro-l-phenylalanine (0.3 mg mL-1). The mixture was vortexed at 60 Hz for 2 min, followed by ultrasonication at ambient temperature for 30 min. Samples were then centrifuged at 13,000 rpm, 4 °C for 15 min. The supernatants were collected using crystal syringes, filtered through 0.22 μm microfilters and transferred to LC vials, followed by storage at −80 °C. LC–MS/MS analyses were performed using an UHPLC system (1290, Agilent Technologies).
Metabolite-transcript correlation analysis
Pearson correlation coefficients (PCCs) were calculated for metabolite and transcript profiles as described previously 41. The mean of all the biological replicates for individual metabolites and the normalized mean value of the transcriptional levels of each gene were evaluated. The coefficients were calculated using the log2 (fold change) values, with PCC>0.90 and PCC P value (PCCP)<0.001 used as the cutoff. The connection network between orthogroups and metabolites was built via Cytoscape (version 3.8.2).
Weighted gene co-expression network analysis
Each orthogroup, out of the 5026 shared by MEM25 and FACHB-9 (including 3101 single copy orthogroups), was designated as a metagene. Taking into account both the transcriptional level and the gene copy number's impact, the metagenes' transcriptional levels were quantified by summing the transcriptional levels of all genes within each orthogroup. The WGCNA version 1.70-3 was used to analyze the transcriptome and metabolome data, with the orthogroups categorized into different modules, correlated with specific trait(s). A cutoff was set with the correlation coefficient > 0.75 and a P-value < 0.005. For the transcriptome, the WGCNA soft threshold of the expression matrix was set at 7, with the scale-free topological model fitting R2 > 0.9. Regarding the metabolome, the values were 9 and 0.9, respectively.
Principal component analysis
Principal component analysis of featured genes was performed using the R package ggord (https://zenodo.org/badge/latestdoi/35334615). The packages prcomp and factoextra were used for the analysis and ggplot2-based visualization. The abscissa of the PCA score chart represents the first principal component, namely PC1, and the ordinate represents the second principal component, namely PC2. The confidence interval level for PCA analysis was set to 0.95.
Machine learning analysis
A machine learning analysis was conducted on a gene family presence-absence matrix across the 46 species to discern crucial genetic traits that differentiate freshwater and marine algae. Each species was represented in binary form, with "1" denoting the presence of a gene family and "0" indicating its absence. Our method involved a two-stage machine learning approach encompassing 86 models and model combinations, including Random Forest, Lasso, glmboost, Ridge, SVM, KNN, NaiveBayes, GBM, DecisionTree, and Enet (with various alpha values). Initially, models performed feature selection to pinpoint potential gene families, followed by additional filtering or classification to enhance classification accuracy. A 5-fold cross-validation method was employed across all models to ensure robust performance evaluation. For models like Ridge Regression without inherent feature constraints, accuracy was incrementally tested with feature sets in batches of 50 genes to determine the optimal feature count for peak performance. This meticulous approach ensured that each model combination achieved its highest classification accuracy, enabling precise identification of genetic traits crucial for distinguishing between freshwater and marine species. The most effective model combination, Random Forest + Elastic Net [0.6], boasting a classification accuracy of 97%, was utilized to highlight featured genes.

For manuscripts utilizing custom algorithms or software that are central to the research but not yet described in published literature, software must be made available to editors and reviewers. We strongly encourage code deposition in a community repository (e.g. GitHub). See the Nature Portfolio guidelines for submitting code & software for further information.

Data

Policy information about availability of data

All manuscripts must include a data availability statement. This statement should provide the following information, where applicable:

- Accession codes, unique identifiers, or web links for publicly available datasets
- A description of any restrictions on data availability
- For clinical datasets or third party data, please ensure that the statement adheres to our policy

All data supporting the findings of this study are publicly available.

Research involving human participants, their data, or biological material

Policy information about studies with human participants or human data. See also policy information about sex, gender (identity/presentation), and sexual orientation and race, ethnicity and racism.

Reporting on sex and gender	Not applicable
Reporting on race, ethnicity, or other socially relevant groupings	Not applicable
Population characteristics	Not applicable
Recruitment	Not applicable

Ethics oversight	Not applicable

Note that full information on the approval of the study protocol must also be provided in the manuscript.

Field-specific reporting

Please select the one below that is the best fit for your research. If you are not sure, read the appropriate sections before making your selection.

☒ Life sciences ☐ Behavioural & social sciences ☐ Ecological, evolutionary & environmental sciences

For a reference copy of the document with all sections, see nature.com/documents/nr-reporting-summary-flat.pdf

Life sciences study design

All studies must disclose on these points even when the disclosure is negative.

Sample size	We have defined the sample size for each result.
Data exclusions	Not applicable
Replication	We have defined the replicates performed and whether they are biological or technical for each result.
Randomization	The methods for sample allocation in the experimental groups has been clearly showed in the legend or main text.
Blinding	The investigators were blinded to group allocation during data collection.

Behavioural & social sciences study design

All studies must disclose on these points even when the disclosure is negative.

Study description	*Briefly describe the study type including whether data are quantitative, qualitative, or mixed-methods (e.g. qualitative cross-sectional, quantitative experimental, mixed-methods case study).*
Research sample	*State the research sample (e.g. Harvard university undergraduates, villagers in rural India) and provide relevant demographic information (e.g. age, sex) and indicate whether the sample is representative. Provide a rationale for the study sample chosen. For studies involving existing datasets, please describe the dataset and source.*
Sampling strategy	*Describe the sampling procedure (e.g. random, snowball, stratified, convenience). Describe the statistical methods that were used to predetermine sample size OR if no sample-size calculation was performed, describe how sample sizes were chosen and provide a rationale for why these sample sizes are sufficient. For qualitative data, please indicate whether data saturation was considered, and what criteria were used to decide that no further sampling was needed.*
Data collection	*Provide details about the data collection procedure, including the instruments or devices used to record the data (e.g. pen and paper, computer, eye tracker, video or audio equipment) whether anyone was present besides the participant(s) and the researcher, and whether the researcher was blind to experimental condition and/or the study hypothesis during data collection.*
Timing	*Indicate the start and stop dates of data collection. If there is a gap between collection periods, state the dates for each sample cohort.*
Data exclusions	*If no data were excluded from the analyses, state so OR if data were excluded, provide the exact number of exclusions and the rationale behind them, indicating whether exclusion criteria were pre-established.*
Non-participation	*State how many participants dropped out/declined participation and the reason(s) given OR provide response rate OR state that no participants dropped out/declined participation.*
Randomization	*If participants were not allocated into experimental groups, state so OR describe how participants were allocated to groups, and if allocation was not random, describe how covariates were controlled.*

Ecological, evolutionary & environmental sciences study design

All studies must disclose on these points even when the disclosure is negative.

Study description	*Briefly describe the study. For quantitative data include treatment factors and interactions, design structure (e.g. factorial, nested, hierarchical), nature and number of experimental units and replicates.*
Research sample	*Describe the research sample (e.g. a group of tagged Passer domesticus, all Stenocereus thurberi within Organ Pipe Cactus National Monument), and provide a rationale for the sample choice. When relevant, describe the organism taxa, source, sex, age range and*

	any manipulations. State what population the sample is meant to represent when applicable. For studies involving existing datasets, describe the data and its source.
Sampling strategy	*Note the sampling procedure. Describe the statistical methods that were used to predetermine sample size OR if no sample-size calculation was performed, describe how sample sizes were chosen and provide a rationale for why these sample sizes are sufficient.*
Data collection	*Describe the data collection procedure, including who recorded the data and how.*
Timing and spatial scale	*Indicate the start and stop dates of data collection, noting the frequency and periodicity of sampling and providing a rationale for these choices. If there is a gap between collection periods, state the dates for each sample cohort. Specify the spatial scale from which the data are taken*
Data exclusions	*If no data were excluded from the analyses, state so OR if data were excluded, describe the exclusions and the rationale behind them, indicating whether exclusion criteria were pre-established.*
Reproducibility	*Describe the measures taken to verify the reproducibility of experimental findings. For each experiment, note whether any attempts to repeat the experiment failed OR state that all attempts to repeat the experiment were successful.*
Randomization	*Describe how samples/organisms/participants were allocated into groups. If allocation was not random, describe how covariates were controlled. If this is not relevant to your study, explain why.*
Blinding	*Describe the extent of blinding used during data acquisition and analysis. If blinding was not possible, describe why OR explain why blinding was not relevant to your study.*

Did the study involve field work? ☐ Yes ☐ No

Field work, collection and transport

Field conditions	*Describe the study conditions for field work, providing relevant parameters (e.g. temperature, rainfall).*
Location	*State the location of the sampling or experiment, providing relevant parameters (e.g. latitude and longitude, elevation, water depth).*
Access & import/export	*Describe the efforts you have made to access habitats and to collect and import/export your samples in a responsible manner and in compliance with local, national and international laws, noting any permits that were obtained (give the name of the issuing authority, the date of issue, and any identifying information).*
Disturbance	*Describe any disturbance caused by the study and how it was minimized.*

Reporting for specific materials, systems and methods

We require information from authors about some types of materials, experimental systems and methods used in many studies. Here, indicate whether each material, system or method listed is relevant to your study. If you are not sure if a list item applies to your research, read the appropriate section before selecting a response.

Materials & experimental systems

n/a	Involved in the study	
☒	☐	Antibodies
☒	☐	Eukaryotic cell lines
☒	☐	Palaeontology and archaeology
☒	☐	Animals and other organisms
☒	☐	Clinical data
☒	☐	Dual use research of concern
☐	☒	Plants

Methods

n/a	Involved in the study	
☒	☐	ChIP-seq
☒	☐	Flow cytometry
☒	☐	MRI-based neuroimaging

Antibodies

Antibodies used	*Describe all antibodies used in the study; as applicable, provide supplier name, catalog number, clone name, and lot number.*
Validation	*Describe the validation of each primary antibody for the species and application, noting any validation statements on the manufacturer's website, relevant citations, antibody profiles in online databases, or data provided in the manuscript.*

Eukaryotic cell lines

Policy information about cell lines and Sex and Gender in Research

Cell line source(s)	*State the source of each cell line used and the sex of all primary cell lines and cells derived from human participants or vertebrate models.*
Authentication	*Describe the authentication procedures for each cell line used OR declare that none of the cell lines used were authenticated.*
Mycoplasma contamination	*Confirm that all cell lines tested negative for mycoplasma contamination OR describe the results of the testing for mycoplasma contamination OR declare that the cell lines were not tested for mycoplasma contamination.*
Commonly misidentified lines (See ICLAC register)	*Name any commonly misidentified cell lines used in the study and provide a rationale for their use.*

Palaeontology and Archaeology

Specimen provenance	*Provide provenance information for specimens and describe permits that were obtained for the work (including the name of the issuing authority, the date of issue, and any identifying information). Permits should encompass collection and, where applicable, export.*
Specimen deposition	*Indicate where the specimens have been deposited to permit free access by other researchers.*
Dating methods	*If new dates are provided, describe how they were obtained (e.g. collection, storage, sample pretreatment and measurement), where they were obtained (i.e. lab name), the calibration program and the protocol for quality assurance OR state that no new dates are provided.*

☐ Tick this box to confirm that the raw and calibrated dates are available in the paper or in Supplementary Information.

Ethics oversight	*Identify the organization(s) that approved or provided guidance on the study protocol, OR state that no ethical approval or guidance was required and explain why not.*

Note that full information on the approval of the study protocol must also be provided in the manuscript.

Animals and other research organisms

Policy information about studies involving animals; ARRIVE guidelines recommended for reporting animal research, and Sex and Gender in Research

Laboratory animals	*For laboratory animals, report species, strain and age OR state that the study did not involve laboratory animals.*
Wild animals	*Provide details on animals observed in or captured in the field; report species and age where possible. Describe how animals were caught and transported and what happened to captive animals after the study (if killed, explain why and describe method; if released, say where and when) OR state that the study did not involve wild animals.*
Reporting on sex	*Indicate if findings apply to only one sex; describe whether sex was considered in study design, methods used for assigning sex. Provide data disaggregated for sex where this information has been collected in the source data as appropriate; provide overall numbers in this Reporting Summary. Please state if this information has not been collected. Report sex-based analyses where performed, justify reasons for lack of sex-based analysis.*
Field-collected samples	*For laboratory work with field-collected samples, describe all relevant parameters such as housing, maintenance, temperature, photoperiod and end-of-experiment protocol OR state that the study did not involve samples collected from the field.*
Ethics oversight	*Identify the organization(s) that approved or provided guidance on the study protocol, OR state that no ethical approval or guidance was required and explain why not.*

Note that full information on the approval of the study protocol must also be provided in the manuscript.

Clinical data

Policy information about clinical studies

All manuscripts should comply with the ICMJE guidelines for publication of clinical research and a completed CONSORT checklist must be included with all submissions.

Clinical trial registration	*Provide the trial registration number from ClinicalTrials.gov or an equivalent agency.*
Study protocol	*Note where the full trial protocol can be accessed OR if not available, explain why.*
Data collection	*Describe the settings and locales of data collection, noting the time periods of recruitment and data collection.*

Outcomes | Describe how you pre-defined primary and secondary outcome measures and how you assessed these measures.

Dual use research of concern

Policy information about dual use research of concern

Hazards

Could the accidental, deliberate or reckless misuse of agents or technologies generated in the work, or the application of information presented in the manuscript, pose a threat to:

No	Yes	
☒	☐	Public health
☒	☐	National security
☒	☐	Crops and/or livestock
☒	☐	Ecosystems
☒	☐	Any other significant area

Experiments of concern

Does the work involve any of these experiments of concern:

No	Yes	
☒	☐	Demonstrate how to render a vaccine ineffective
☒	☐	Confer resistance to therapeutically useful antibiotics or antiviral agents
☒	☐	Enhance the virulence of a pathogen or render a nonpathogen virulent
☒	☐	Increase transmissibility of a pathogen
☒	☐	Alter the host range of a pathogen
☒	☐	Enable evasion of diagnostic/detection modalities
☒	☐	Enable the weaponization of a biological agent or toxin
☒	☐	Any other potentially harmful combination of experiments and agents

Plants

Seed stocks	A. thaliana Col-0 (WT) and SALK_202085C (cnbp) T-DNA insertion mutant lines were obtained from The Nottingham Arabidopsis Stock Centre (NASC).
Novel plant genotypes	Cas9-mediated knockout Nannochlorpsis lines targeting the homologs of CP4g4492, CP1g58, CP5g5156, CP8g6201, CP11g8082, and CP10g8454.
Authentication	Cas9-mediated target gene disruption in Nannochloropsis was conducted using an episomal CRISPR system. Mutations with target disruption were screened by PCR with specific primers (Supplementary Data 18). The growth of microalgae was monitored by measuring the turbidity or cell number at specified intervals with a Gene Quant 1300 Spectrophotometer (GE) or LUNA-II™ Automated Cell Counter (Logos Biosystems). Dry cell weight of a 10-ml algal culture was determined simultaneously.

ChIP-seq

Data deposition

☐ Confirm that both raw and final processed data have been deposited in a public database such as GEO.

☐ Confirm that you have deposited or provided access to graph files (e.g. BED files) for the called peaks.

Data access links<br>*May remain private before publication.*	*For "Initial submission" or "Revised version" documents, provide reviewer access links. For your "Final submission" document, provide a link to the deposited data.*
Files in database submission	*Provide a list of all files available in the database submission.*
Genome browser session<br>(e.g. UCSC)	*Provide a link to an anonymized genome browser session for "Initial submission" and "Revised version" documents only, to enable peer review. Write "no longer applicable" for "Final submission" documents.*

Methodology

Replicates	Describe the experimental replicates, specifying number, type and replicate agreement.
Sequencing depth	Describe the sequencing depth for each experiment, providing the total number of reads, uniquely mapped reads, length of reads and whether they were paired- or single-end.
Antibodies	Describe the antibodies used for the ChIP-seq experiments; as applicable, provide supplier name, catalog number, clone name, and lot number.
Peak calling parameters	Specify the command line program and parameters used for read mapping and peak calling, including the ChIP, control and index files used.
Data quality	Describe the methods used to ensure data quality in full detail, including how many peaks are at FDR 5% and above 5-fold enrichment.
Software	Describe the software used to collect and analyze the ChIP-seq data. For custom code that has been deposited into a community repository, provide accession details.

Flow Cytometry

Plots

Confirm that:

- [ ] The axis labels state the marker and fluorochrome used (e.g. CD4-FITC).
- [ ] The axis scales are clearly visible. Include numbers along axes only for bottom left plot of group (a 'group' is an analysis of identical markers).
- [ ] All plots are contour plots with outliers or pseudocolor plots.
- [ ] A numerical value for number of cells or percentage (with statistics) is provided.

Methodology

Sample preparation	Describe the sample preparation, detailing the biological source of the cells and any tissue processing steps used.
Instrument	Identify the instrument used for data collection, specifying make and model number.
Software	Describe the software used to collect and analyze the flow cytometry data. For custom code that has been deposited into a community repository, provide accession details.
Cell population abundance	Describe the abundance of the relevant cell populations within post-sort fractions, providing details on the purity of the samples and how it was determined.
Gating strategy	Describe the gating strategy used for all relevant experiments, specifying the preliminary FSC/SSC gates of the starting cell population, indicating where boundaries between "positive" and "negative" staining cell populations are defined.

- [ ] Tick this box to confirm that a figure exemplifying the gating strategy is provided in the Supplementary Information.

Magnetic resonance imaging

Experimental design

Design type	Indicate task or resting state; event-related or block design.
Design specifications	Specify the number of blocks, trials or experimental units per session and/or subject, and specify the length of each trial or block (if trials are blocked) and interval between trials.
Behavioral performance measures	State number and/or type of variables recorded (e.g. correct button press, response time) and what statistics were used to establish that the subjects were performing the task as expected (e.g. mean, range, and/or standard deviation across subjects).

Acquisition

Imaging type(s) | *Specify: functional, structural, diffusion, perfusion.*

Field strength | *Specify in Tesla*

Sequence & imaging parameters | *Specify the pulse sequence type (gradient echo, spin echo, etc.), imaging type (EPI, spiral, etc.), field of view, matrix size, slice thickness, orientation and TE/TR/flip angle.*

Area of acquisition | *State whether a whole brain scan was used OR define the area of acquisition, describing how the region was determined.*

Diffusion MRI ☐ Used ☐ Not used

Preprocessing

Preprocessing software | *Provide detail on software version and revision number and on specific parameters (model/functions, brain extraction, segmentation, smoothing kernel size, etc.).*

Normalization | *If data were normalized/standardized, describe the approach(es): specify linear or non-linear and define image types used for transformation OR indicate that data were not normalized and explain rationale for lack of normalization.*

Normalization template | *Describe the template used for normalization/transformation, specifying subject space or group standardized space (e.g. original Talairach, MNI305, ICBM152) OR indicate that the data were not normalized.*

Noise and artifact removal | *Describe your procedure(s) for artifact and structured noise removal, specifying motion parameters, tissue signals and physiological signals (heart rate, respiration).*

Volume censoring | *Define your software and/or method and criteria for volume censoring, and state the extent of such censoring.*

Statistical modeling & inference

Model type and settings | *Specify type (mass univariate, multivariate, RSA, predictive, etc.) and describe essential details of the model at the first and second levels (e.g. fixed, random or mixed effects; drift or auto-correlation).*

Effect(s) tested | *Define precise effect in terms of the task or stimulus conditions instead of psychological concepts and indicate whether ANOVA or factorial designs were used.*

Specify type of analysis: ☐ Whole brain ☐ ROI-based ☐ Both

Statistic type for inference | *Specify voxel-wise or cluster-wise and report all relevant parameters for cluster-wise methods.*

(See Eklund et al. 2016)

Correction | *Describe the type of correction and how it is obtained for multiple comparisons (e.g. FWE, FDR, permutation or Monte Carlo).*

Models & analysis

n/a	Involved in the study
☐	☐ Functional and/or effective connectivity
☐	☐ Graph analysis
☐	☐ Multivariate modeling or predictive analysis

Functional and/or effective connectivity | *Report the measures of dependence used and the model details (e.g. Pearson correlation, partial correlation, mutual information).*

Graph analysis | *Report the dependent variable and connectivity measure, specifying weighted graph or binarized graph, subject- or group-level, and the global and/or node summaries used (e.g. clustering coefficient, efficiency, etc.).*

Multivariate modeling and predictive analysis | *Specify independent variables, features extraction and dimension reduction, model, training and evaluation metrics.*
