# Supplementary figures and images for: Cross-species dissection of saline-related genes by genetically deciphering a euryhaline microalga Chlorella sp
Source: Nat Commun. 2026 Jan 13;17:1577. doi: 10.1038/s41467-026-68287-6 (PMC12902096; doi:10.1038/s41467-026-68287-6)

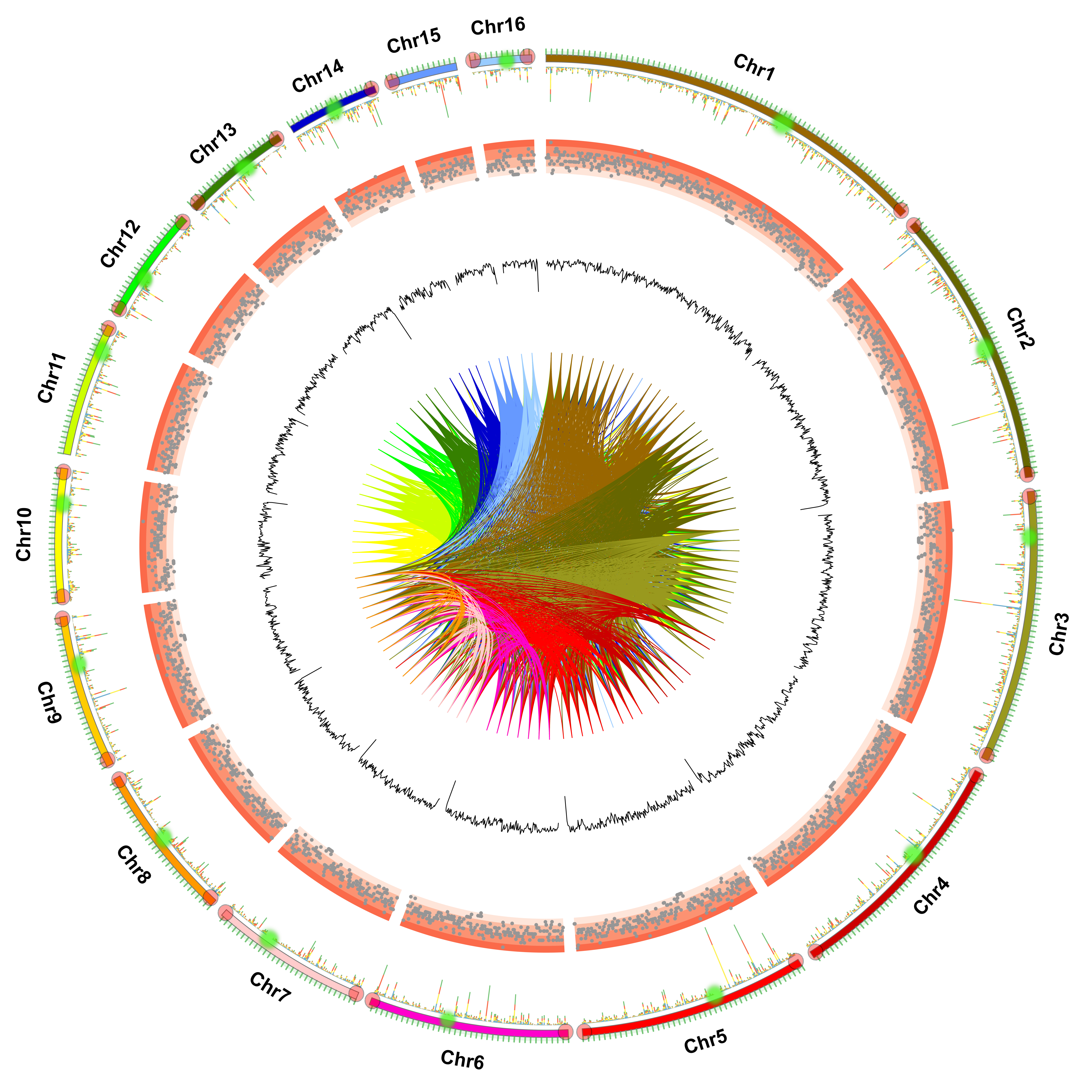

Supplement: Supplementary file 6 — Source Data [file 41467_2026_68287_MOESM6_ESM.zip › 8-Source_Data_and_code/Fig1_data_and_code/Fig1a_circos/Fig1a.png]

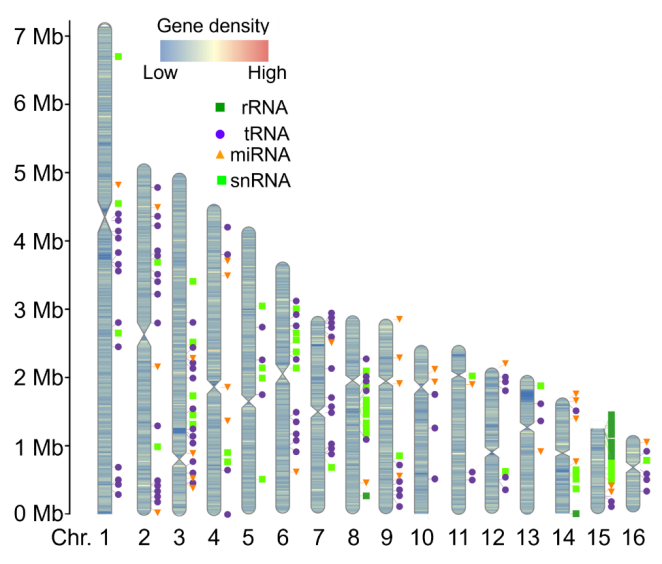

Supplement: Supplementary file 6 — Source Data [file 41467_2026_68287_MOESM6_ESM.zip › 8-Source_Data_and_code/Fig1_data_and_code/Fig1b_karyotype/Fig1b.png]

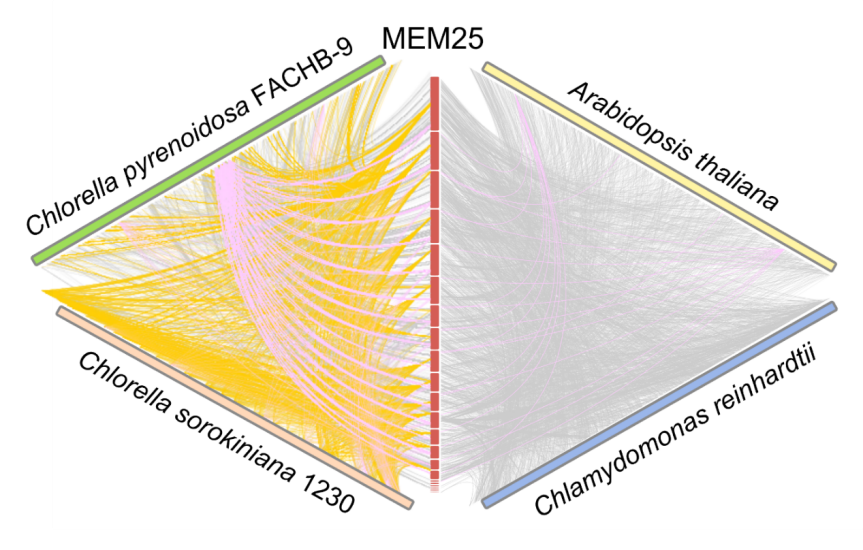

Supplement: Supplementary file 6 — Source Data [file 41467_2026_68287_MOESM6_ESM.zip › 8-Source_Data_and_code/Fig1_data_and_code/Fig1c_synteny/Fig1c.png]

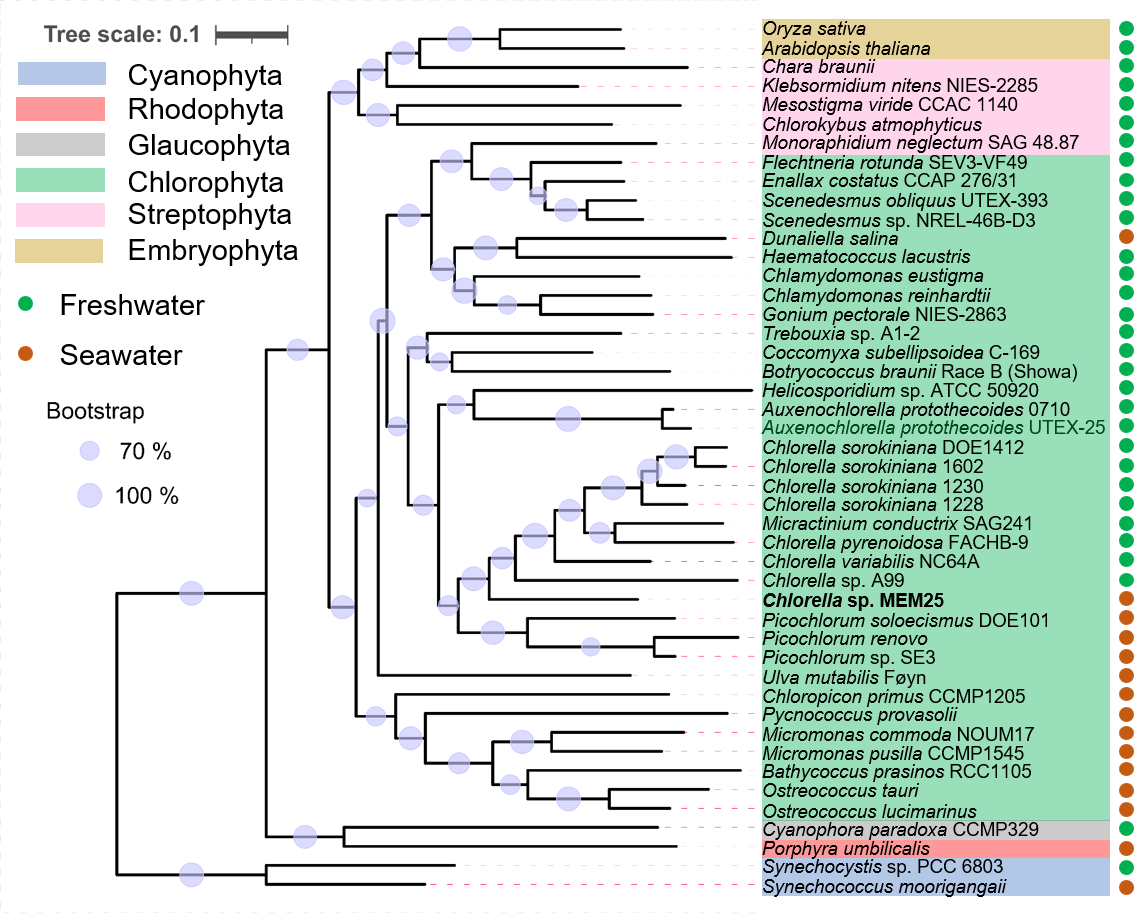

Supplement: Supplementary file 6 — Source Data [file 41467_2026_68287_MOESM6_ESM.zip › 8-Source_Data_and_code/Fig2_data_and_code/Fig2.png]

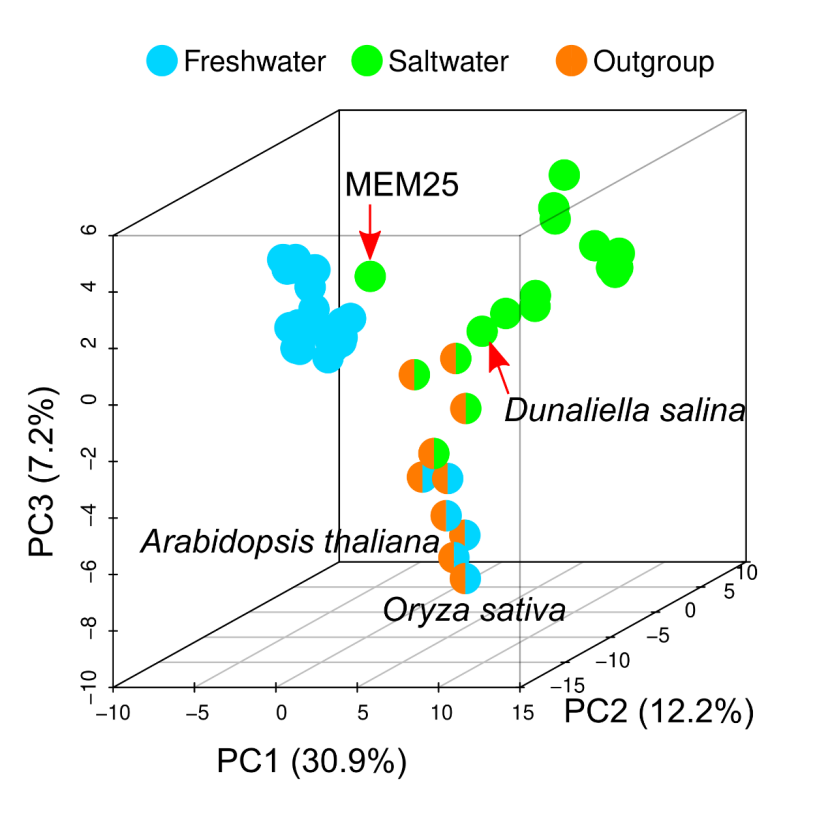

Supplement: Supplementary file 6 — Source Data [file 41467_2026_68287_MOESM6_ESM.zip › 8-Source_Data_and_code/Fig3_data_and_code/Fig3.png]

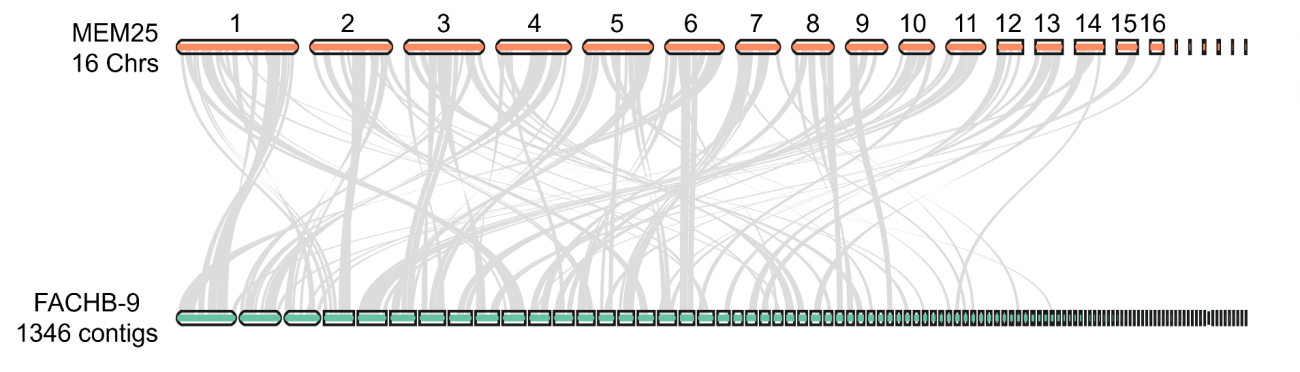

Supplement: Supplementary file 6 — Source Data [file 41467_2026_68287_MOESM6_ESM.zip › 8-Source_Data_and_code/Fig4_data_and_code/Fig4a/Fig4a.png]

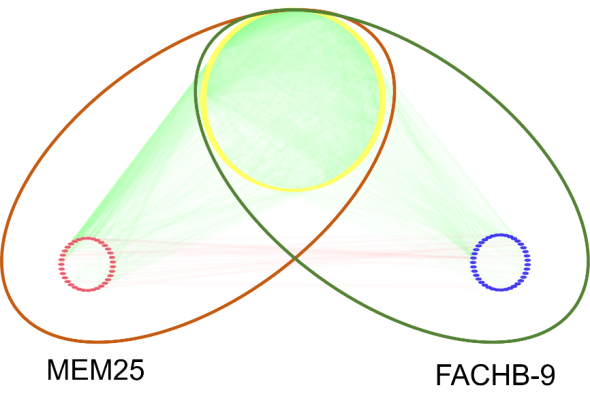

Supplement: Supplementary file 6 — Source Data [file 41467_2026_68287_MOESM6_ESM.zip › 8-Source_Data_and_code/Fig4_data_and_code/Fig4b/Fig4b.png]

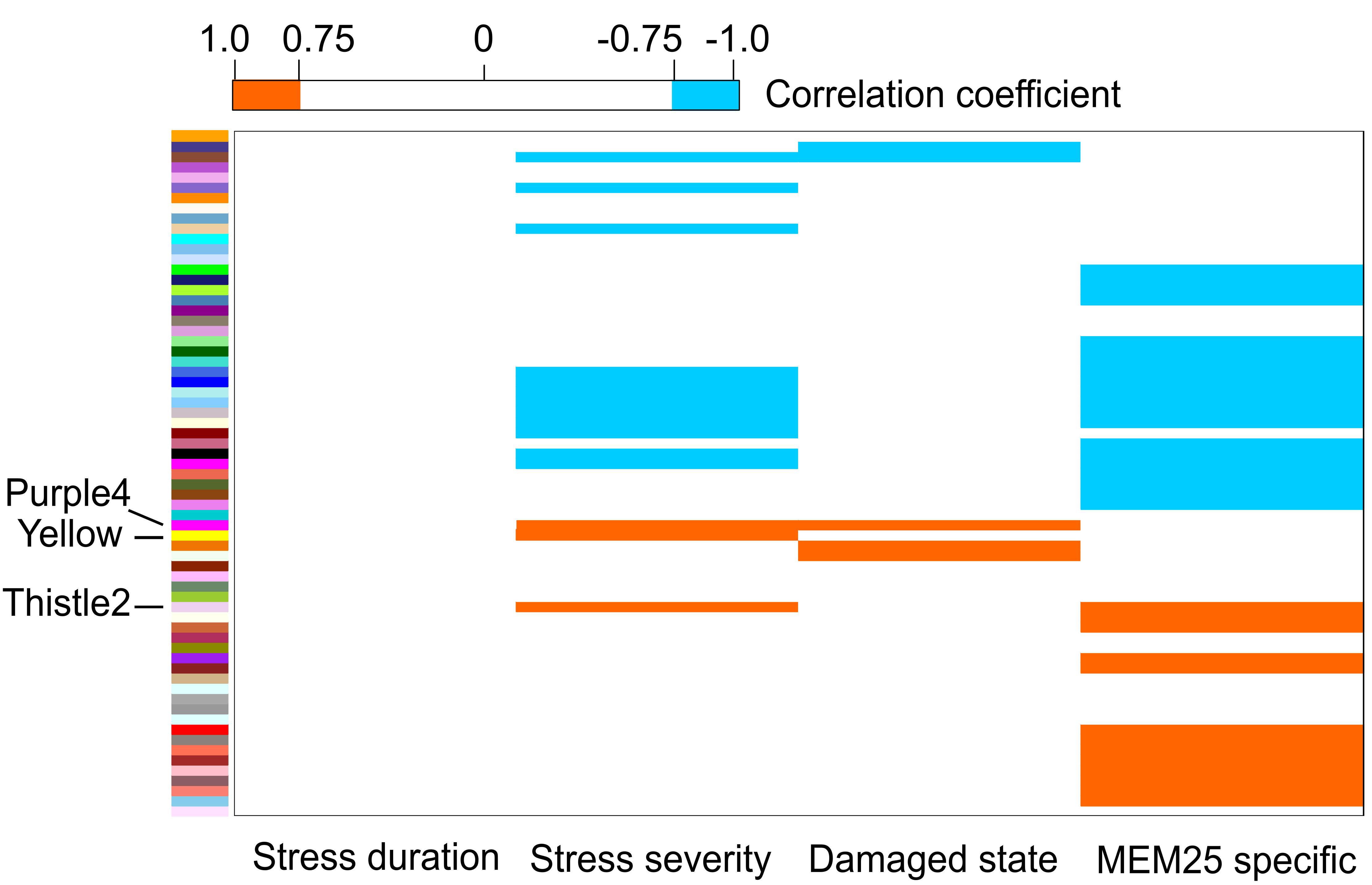

Supplement: Supplementary file 6 — Source Data [file 41467_2026_68287_MOESM6_ESM.zip › 8-Source_Data_and_code/Fig4_data_and_code/Fig4c/Fig4c.png]

### Scale independence

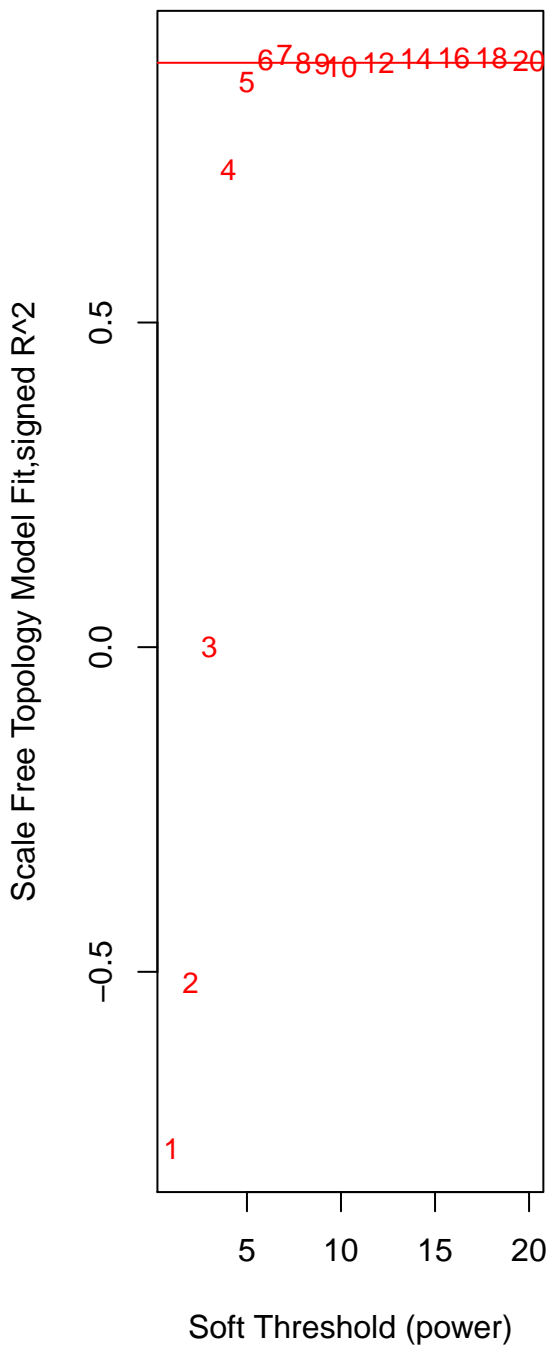

### Mean connectivity

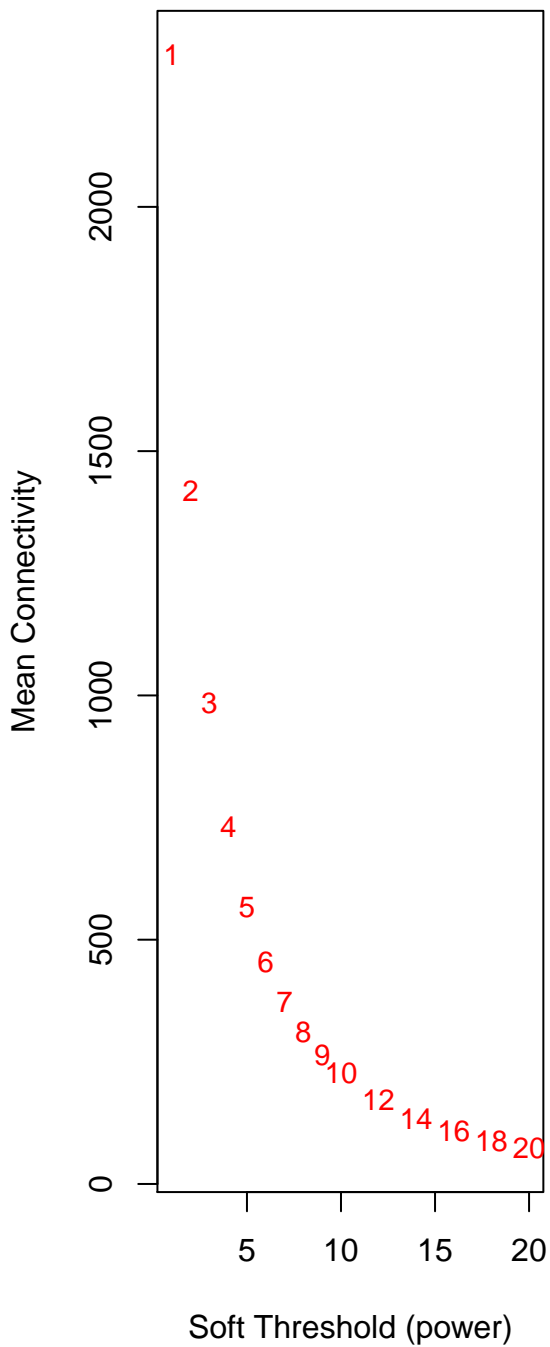

Supplement: Supplementary file 6 — Source Data [file 41467_2026_68287_MOESM6_ESM.zip › 8-Source_Data_and_code/Fig4_data_and_code/Fig4c/power_WGCNA.pdf]

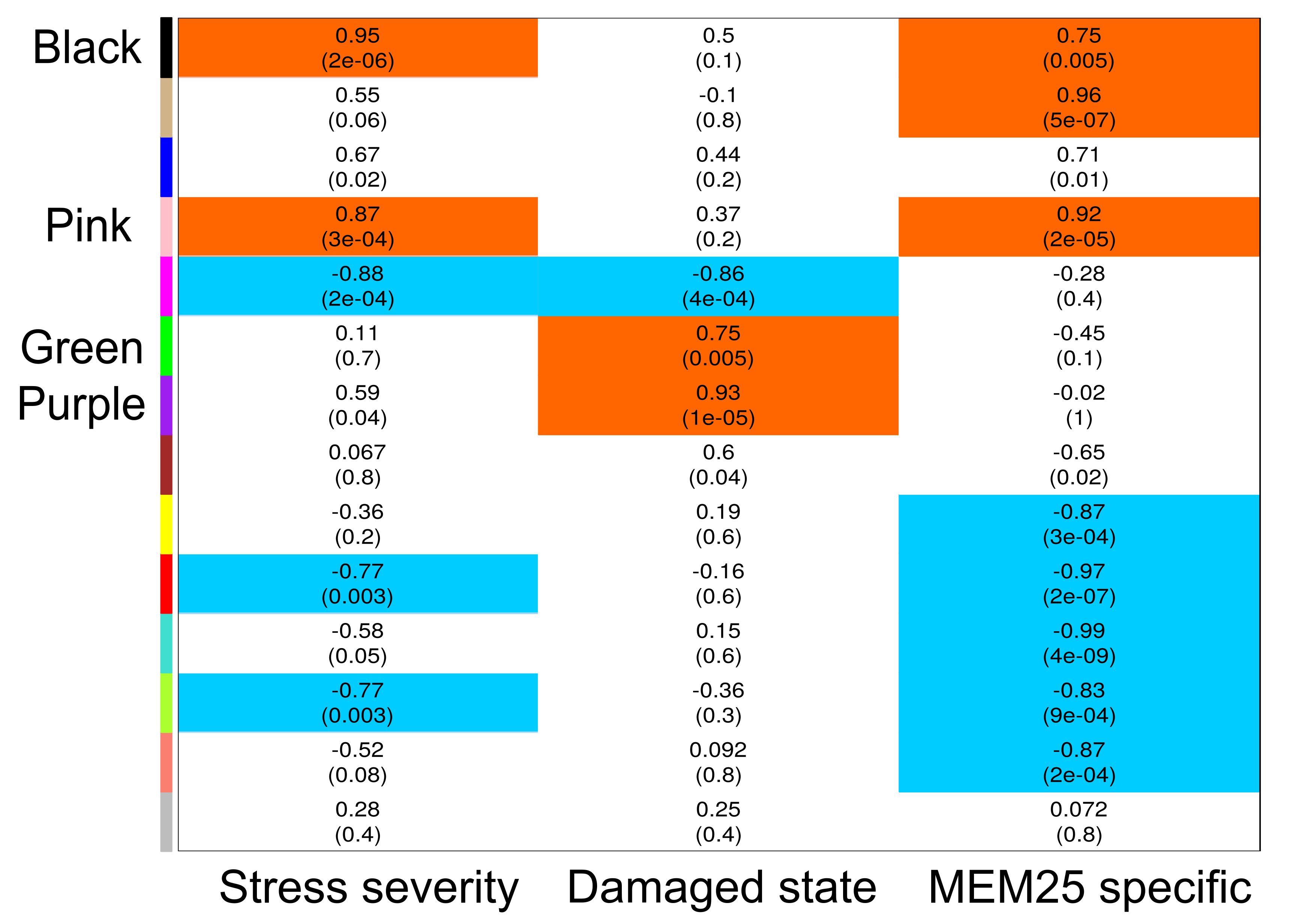

Supplement: Supplementary file 6 — Source Data [file 41467_2026_68287_MOESM6_ESM.zip › 8-Source_Data_and_code/Fig4_data_and_code/Fig4d/Fig4d.png]

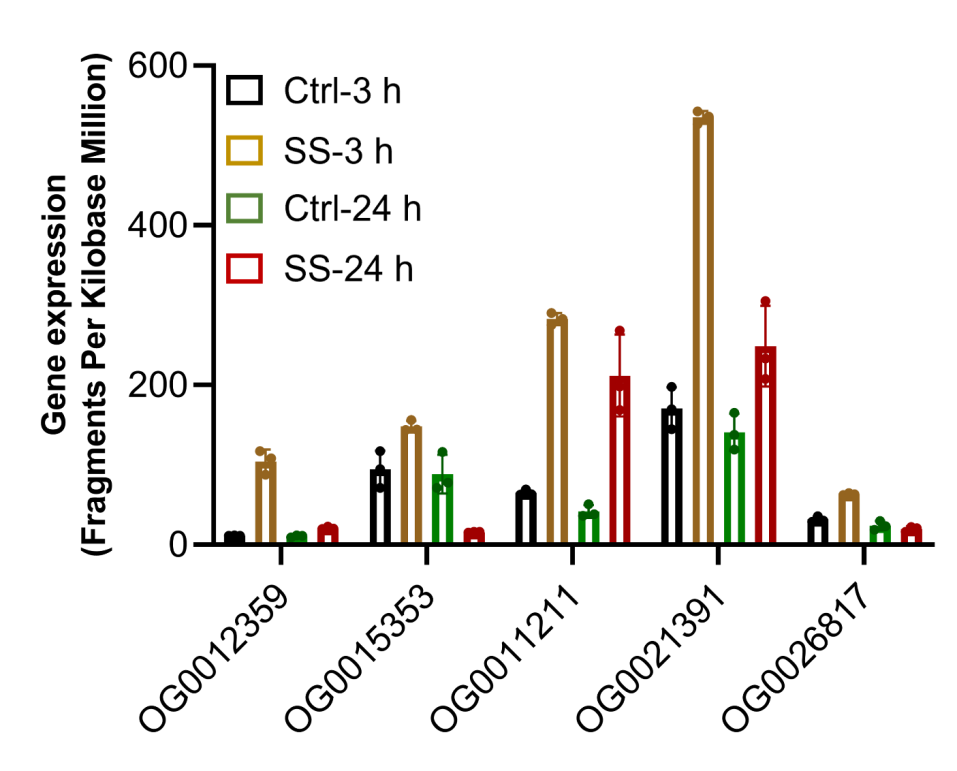

Supplement: Supplementary file 6 — Source Data [file 41467_2026_68287_MOESM6_ESM.zip › 8-Source_Data_and_code/Fig5_data_and_code/Fig5a/Fig5a.png]

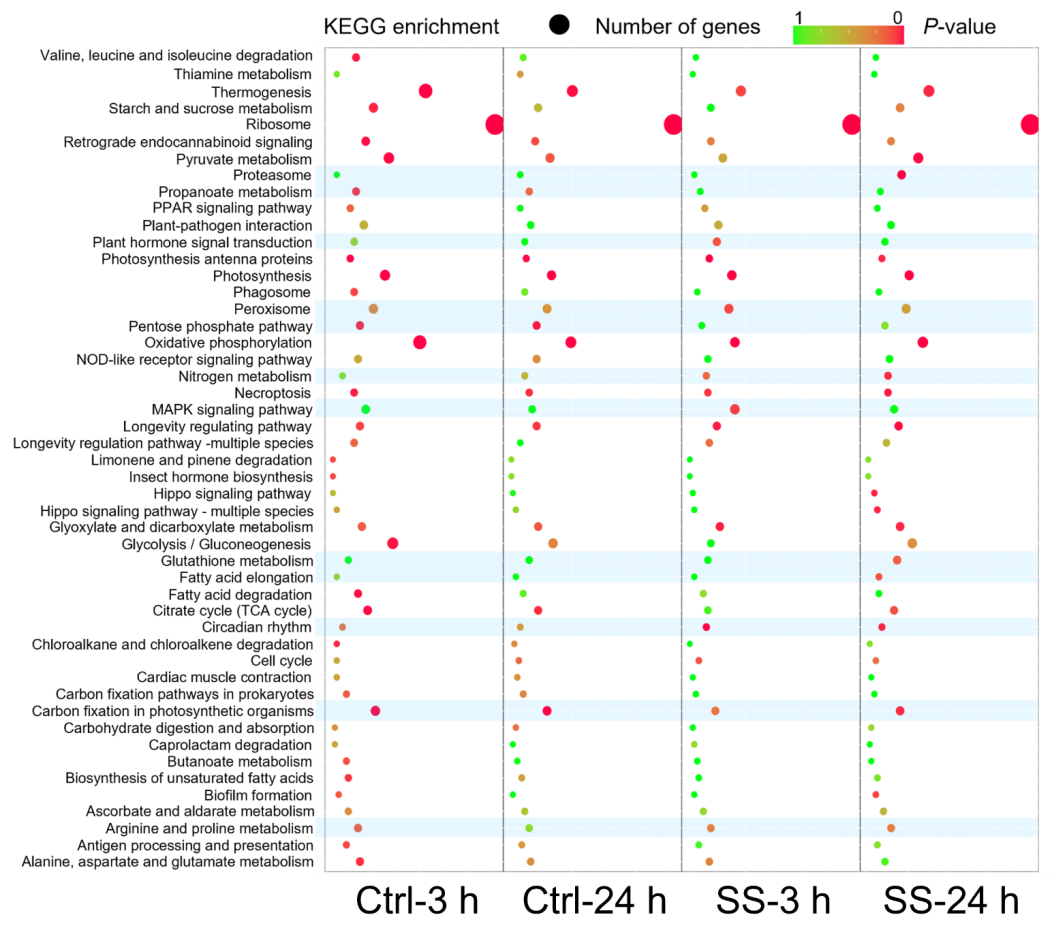

Supplement: Supplementary file 6 — Source Data [file 41467_2026_68287_MOESM6_ESM.zip › 8-Source_Data_and_code/Fig5_data_and_code/Fig5b/Fig5b.png]

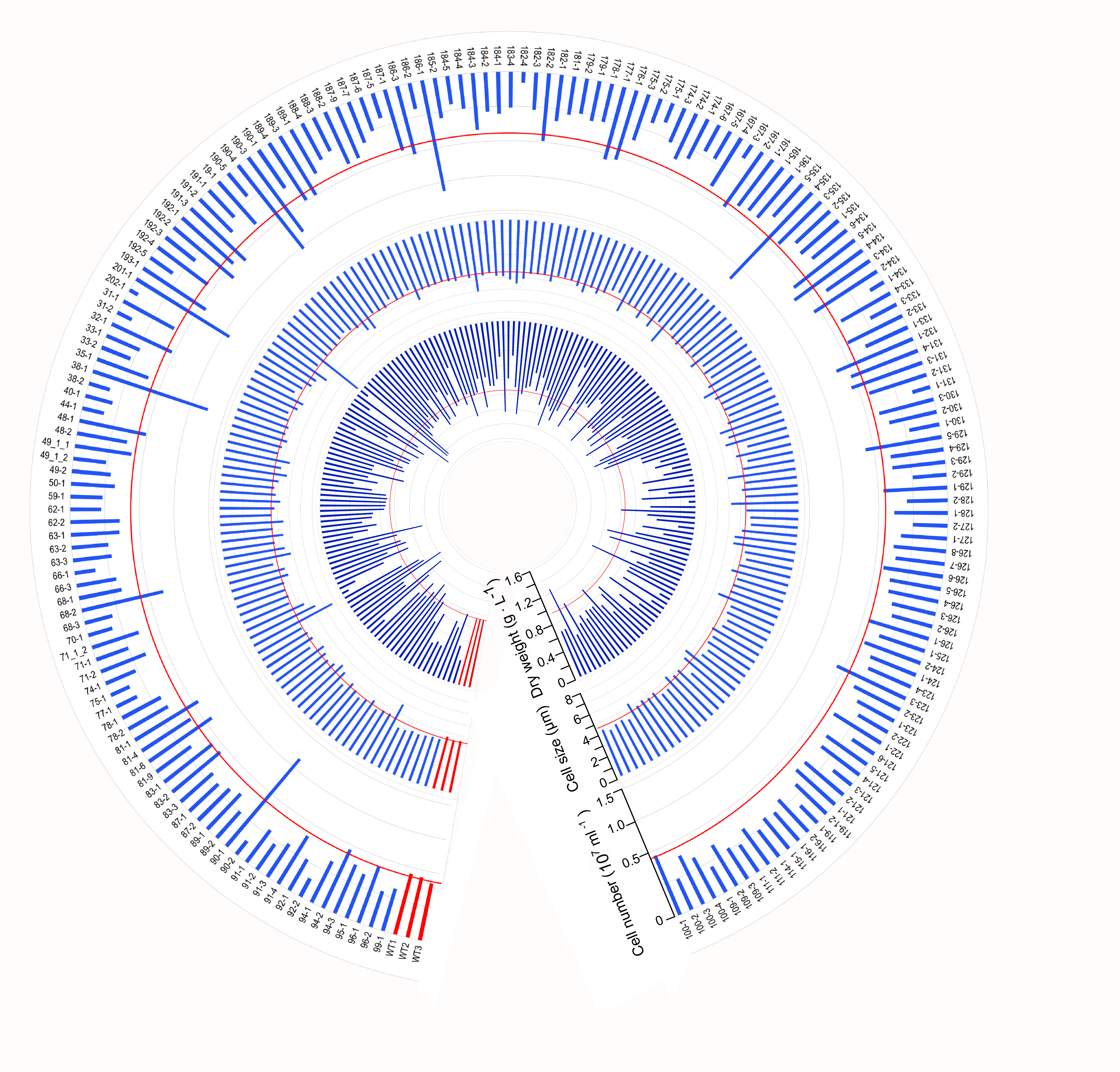

Supplement: Supplementary file 6 — Source Data [file 41467_2026_68287_MOESM6_ESM.zip › 8-Source_Data_and_code/Fig6_data_and_code/Fig6a/Fig6a.png]

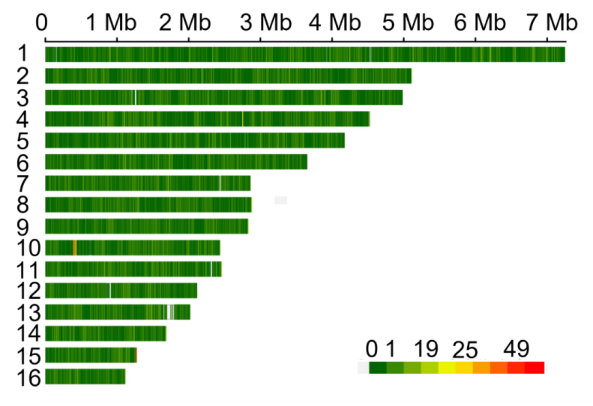

Supplement: Supplementary file 6 — Source Data [file 41467_2026_68287_MOESM6_ESM.zip › 8-Source_Data_and_code/Fig6_data_and_code/Fig6b/Fig6b.png]

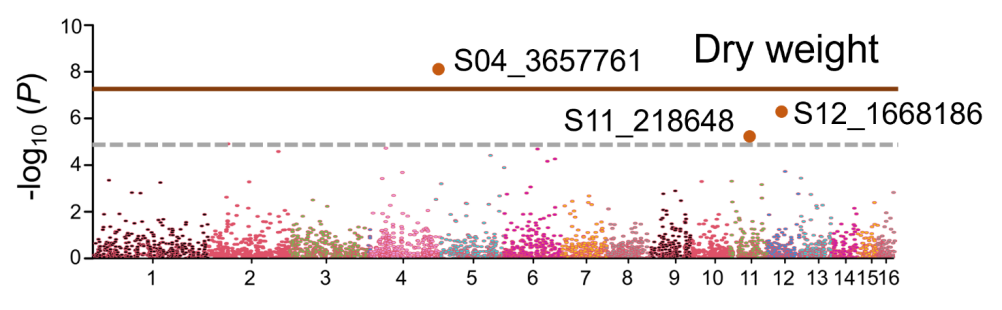

Supplement: Supplementary file 6 — Source Data [file 41467_2026_68287_MOESM6_ESM.zip › 8-Source_Data_and_code/Fig6_data_and_code/Fig6c/Fig6c.png]

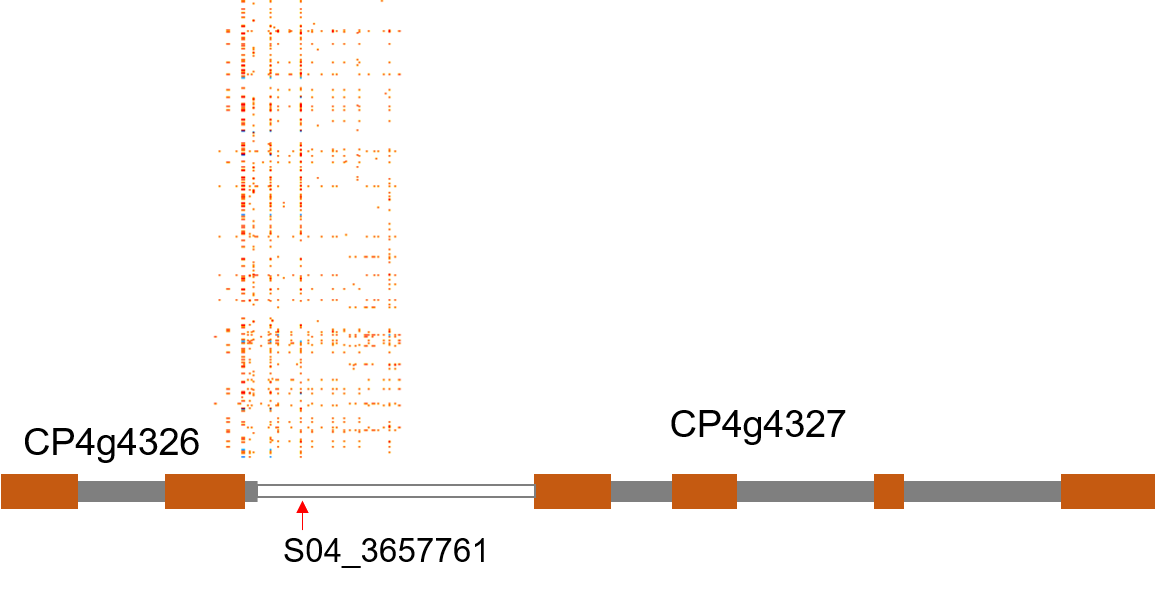

Supplement: Supplementary file 6 — Source Data [file 41467_2026_68287_MOESM6_ESM.zip › 8-Source_Data_and_code/Fig6_data_and_code/Fig6d/Fig6d.png]

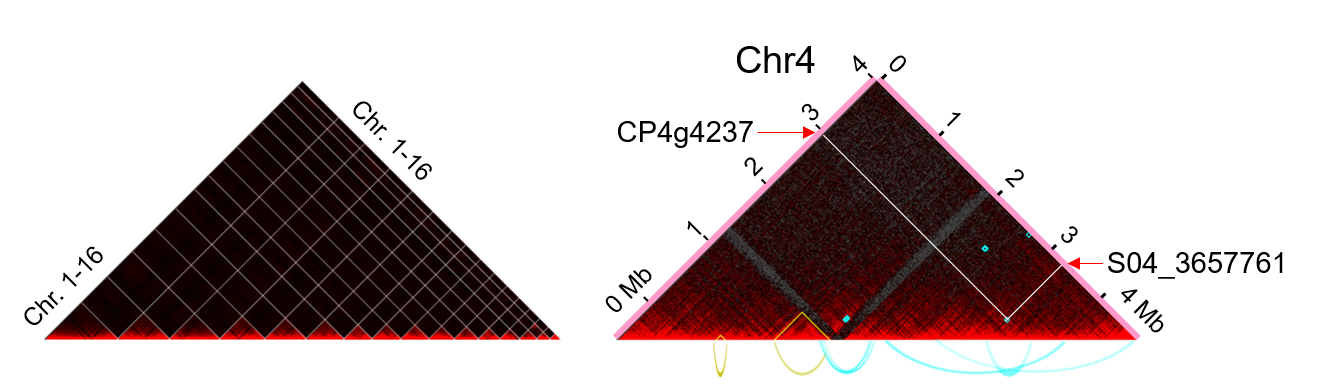

Supplement: Supplementary file 6 — Source Data [file 41467_2026_68287_MOESM6_ESM.zip › 8-Source_Data_and_code/Fig6_data_and_code/Fig6e/Fig6e.png]

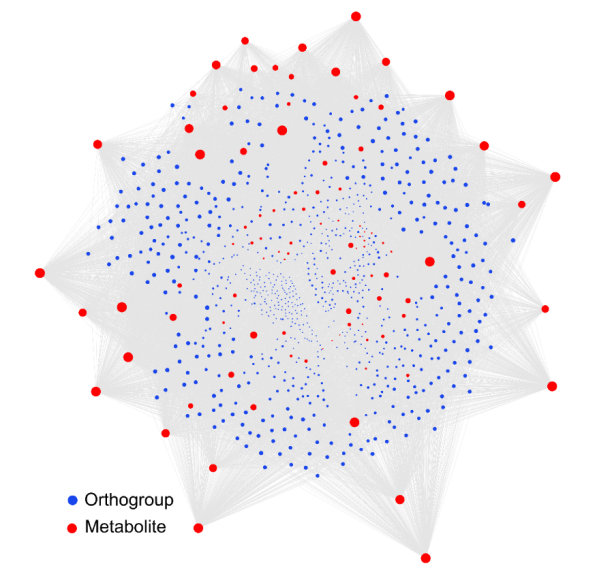

Supplement: Supplementary file 6 — Source Data [file 41467_2026_68287_MOESM6_ESM.zip › 8-Source_Data_and_code/Fig7_data_and_code/Fig7a/Fig7a.png]

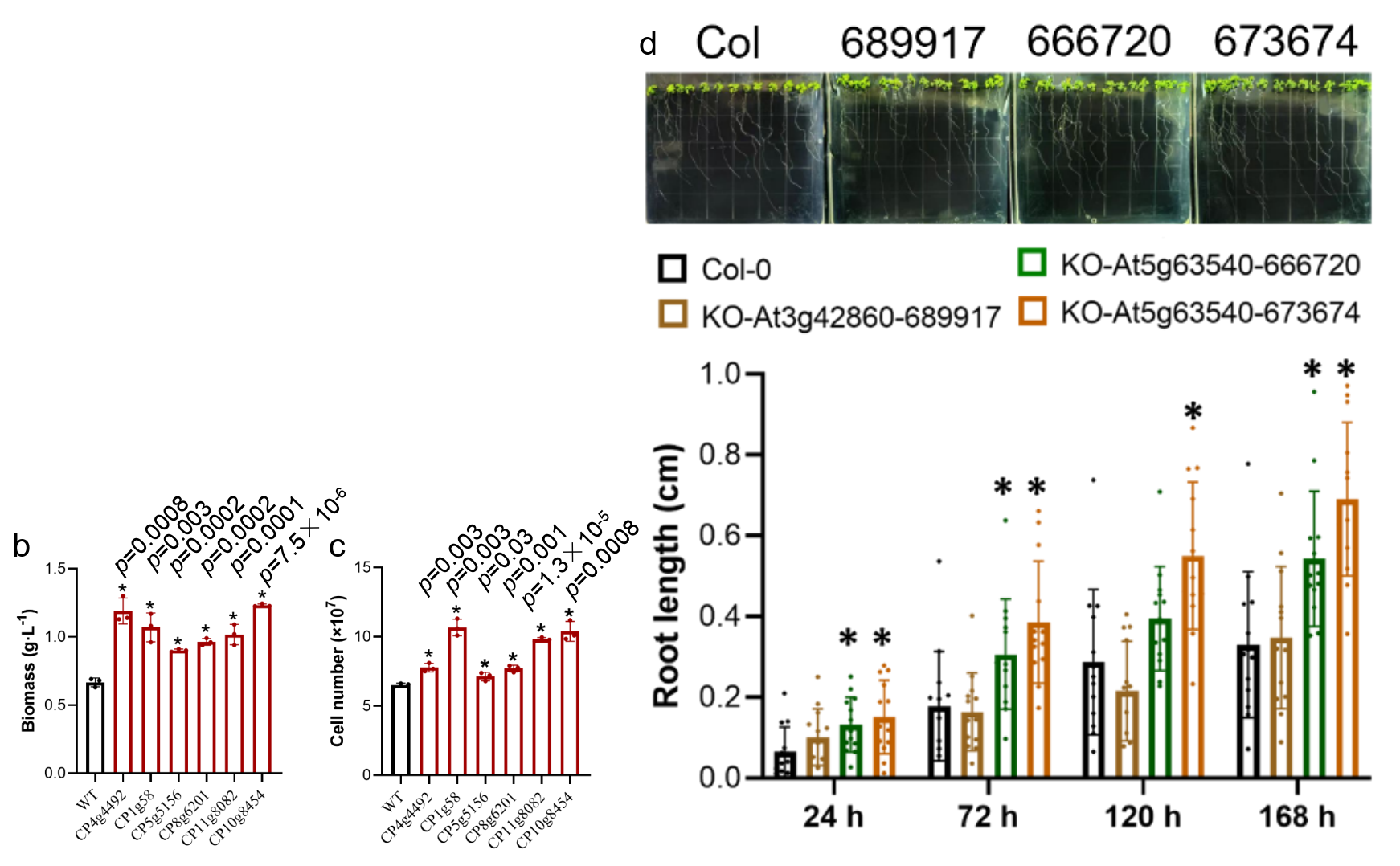

Supplement: Supplementary file 6 — Source Data [file 41467_2026_68287_MOESM6_ESM.zip › 8-Source_Data_and_code/Fig7_data_and_code/Fig7b,c,d/Fig7bcd.png]

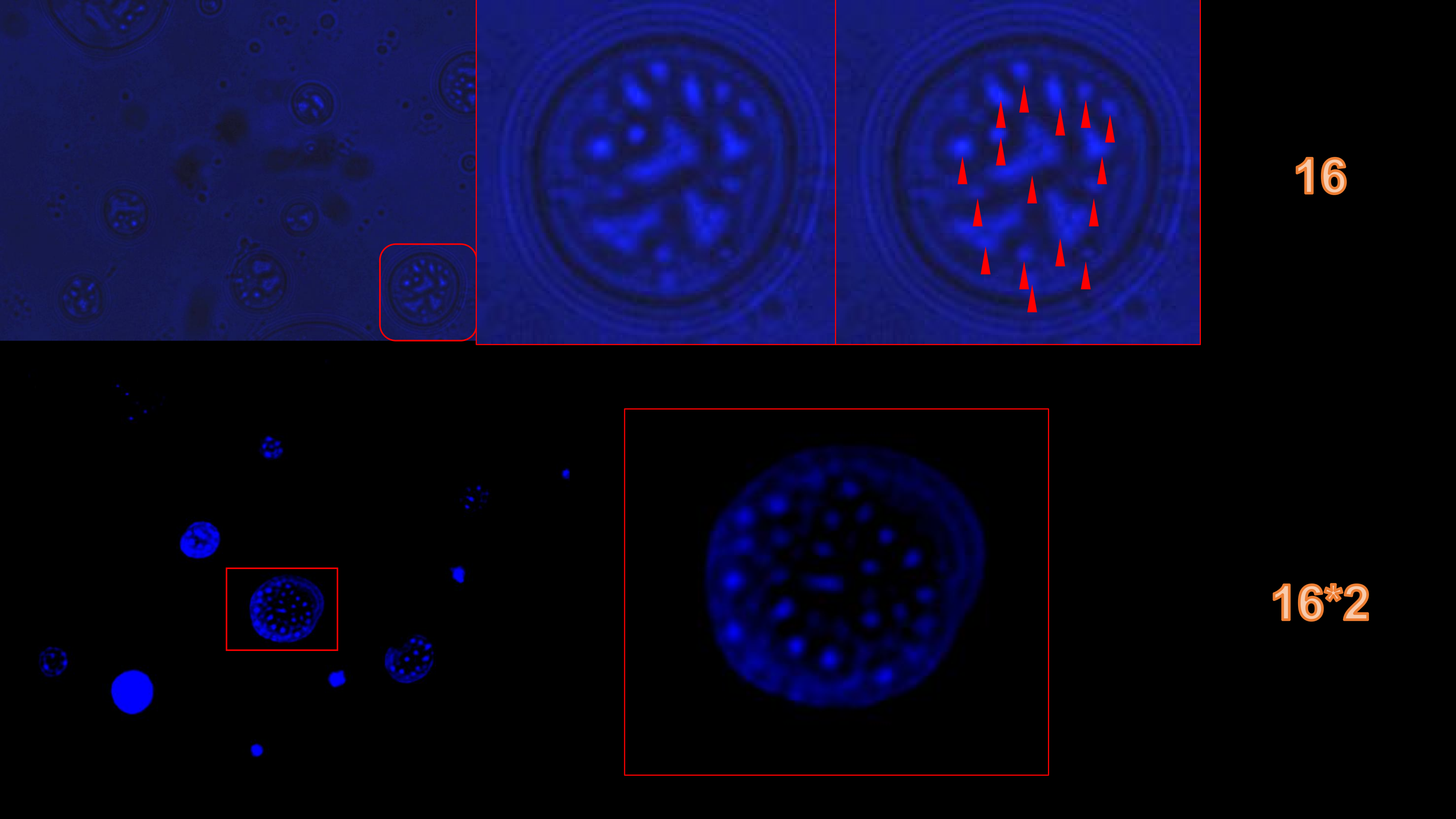

Supplement: Supplementary file 6 — Source Data [file 41467_2026_68287_MOESM6_ESM.zip › 8-Source_Data_and_code/Original_Data_for_Supplementary_Figure/Fig_S1_DAPI.png]
